# Self‐Extracting Dextran‐Based Hydrogel Microneedle Arrays with an Interpenetrating Bioelectroenzymatic Sensor for Transdermal Monitoring with Matrix Protection

**DOI:** 10.1002/adhm.202403209

**Published:** 2024-11-24

**Authors:** Bastien Darmau, Marta Sacchi, Isabelle Texier, Andrew J. Gross

**Affiliations:** ^1^ Department of Molecular Chemistry Univ. Grenoble Alpes‐CNRS 38041 Grenoble France; ^2^ CEA LETI Univ. Grenoble Alpes F‐38054 Grenoble France

**Keywords:** biopolymers, continuous glucose monitoring, electrochemical sensors, in vivo bioelectrocatalysis, wearable biosensors

## Abstract

Continuous glucose monitors have revolutionized diabetes management, yet such devices are limited by their cost, invasiveness, and stability. Microneedle (MN) arrays could offer improved comfort compared to invasive implanted or mm‐sized needle devices, but such arrays are hampered by complex fabrication processes, limited mechanical and sensor stability, and/or cytotoxicity concerns. This work demonstrates the first crosslinked hydrogel microneedle‐bioelectroenzymatic sensor arrays capable of biomarker extraction and robust transdermal continuous monitoring in artificial interstitial fluid for 10 days. The fabrication process via micromolding of dextran‐methacrylate (Dex‐MA) and dry‐state visible light crosslinking is simple and permits the robust fixation of diverse prefabricated electrodes in a single array. Dry‐state crosslinking minimized material shrinkage to enable the formation of resistant Dex‐MA microneedles with shape control and reproducibility. The polymer substitution level (9–62%) and mass content (10–30 wt%) affect the mechanical, swelling, and bioelectrocatalytic properties of the integrated sensors. Crosslinked Dex‐MA hydrogel matrices provide beneficial cytotoxicity protection and flux‐limiting membrane properties to the integrated second generation dehydrogenase‐based nanostructured buckypaper biosensor and Ag/AgCl reference electrodes. Polysaccharide‐based microneedle technology with encapsulated porous bioelectrodes promise to be a valuable alternative to more invasive devices for safer and longer‐term biomarker monitoring.

## Graphical abstract

A polysaccharide‐based microneedle platform is reported towards safer and longer‐term continuous transdermal biomarker monitoring beyond 10 days. The photocrosslinked dextran‐methacrylate hydrogel microneedles enable artificial skin penetration and interstitial fluid extraction to the hydrogel‐encapsulated bioelectroenzymatic glucose sensor. The hydrogel microneedle sensor benefits from polymer interpenetration, cytotoxicity protection, and flux‐limiting membrane properties provided by the chemically crosslinked dextran matrix.

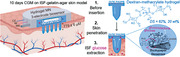

## 1 Introduction

Next‐generation wearable sensors capable of minimally invasive long‐term monitoring of biomarkers promise to substantially improve personalized medicine, telehealth and digital therapeutics.^[^ ^1^ ^]^ Commercial enzyme‐based electrochemical biosensors that exploit glucose‐oxidizing redox enzymes such as glucose oxidase (GOx) and flavin‐dependent glucose dehydrogenase (FAD‐GDH) to drive signal transduction have revolutionized the management and treatment of diabetes.^[^ ^2^, ^3^ ^]^ GOx is the gold standard enzyme while FAD‐GDH has emerged more recently due to advantages such as insensitivity to oxygen (in contrast to GOx) and high substrate selectivity and reactivity. Enzymes offer attractive prospects for sensing such as high reactivity under mild conditions and exquisite selectivity for biomolecules.^[^ ^4^ ^]^ Nevertheless, enzymes and enzyme‐electrode interfaces have deficiencies relating to their stability and activity that greatly limit their practical use, especially for continuous monitoring in vivo. Substantial advancements in protein engineering,^[^ ^5^, ^6^ ^]^ electrode design with nanomaterials such as carbon nanotubes (CNTs)^[^ ^7^, ^8^, ^9^ ^]^ and hierarchically meso/macro‐porous carbons,^[^ ^10^, ^11^, ^12^ ^]^ and electrical enzyme “wiring” strategies,^[^ ^9^, ^13^ ^]^ are helping to address these limitations. Advanced porous electrodes with nanostructured surfaces and sophisticated surface chemistries have become essential for improving mediated and direct electron transfer (MET and DET) reactions for second and third generation biosensing and monitoring.^[^ ^13^ ^]^

State‐of‐the‐art wearable continuous glucose monitoring (CGM) devices are capable of quasi‐continuous monitoring in vivo in subcutaneous interstitial fluid (ISF) for up to 7–14 days. Relevant examples of commercial CGM devices include the Abbot Freestyle Libre, Medtronic Guardian, and Dexcom G series that employ GOx as the glucose‐oxidizing enzyme. Currently, CGM devices have limited sensor lifetime, stability, and precision, and rely on 5–11 mm long invasive needles to access the ISF.^[^ ^14^ ^]^ Monitoring is performed in the deeper hypodermis layer, also known as the subcutaneous fat layer, that is not ideal for biomarker monitoring due to factors such as its variable cellular composition and low fluid content.^[^ ^15^, ^16^ ^]^ For example, the high content of adipocytes (fat cells) as well as nerves, fibroblasts and other connective cells can hinder biomarker mass transport and fluid flow, affecting response times and reliability. In contrast, the dermis has a lower cellular content, higher ISF content, proximity to capillaries, and is less deep in the skin structure. The development of CGM devices capable of monitoring in dermis is therefore of interest.

ISF has established diagnostic value for diverse biomarkers with excellent correlation to blood glucose levels.^[^ ^14^, ^16^, ^17^ ^]^ While many analytes may correlate well, particularly low molecular weight species with high diffusivity, this is not necessarily the case for larger molecules and proteins that can be more problematic, for example, due to paracellular transport filtering effects.^[^ ^16^, ^17^ ^]^ Electrochemical biosensor research is largely focused on glucose for practical and financial reasons. There is also significant interest in the development of sensors for the monitoring of biomarkers such as lactate,^[^ ^18^ ^]^ ketones,^[^ ^19^ ^]^ and nitrate,^[^ ^20^ ^]^ due to their diagnostic value. Future second and third generation body‐integrated biosensors, relying on MET and DET mechanisms, respectively, should provide improved comfort, safety, and analytical performance.

Microneedle (MN)‐based electrochemical sensors with needle heights of 20–2000 µm may provide the solution to the use of more invasive implantable and wearable mm‐sized needle‐based devices.^[^ ^21^, ^22^ ^]^ MN sensors are also an attractive alternative to noninvasive reverse iontophoresis devices that extract biomarkers via electroosmosis with limitations such as discomfort and extraction efficiency.^[^ ^23^, ^24^ ^]^ MN arrays can penetrate the stratum corneum and the viable epidermis without reaching nerve endings or capillary blood vessels, for painless access to dermal ISF. MN biosensors have been developed for in situ analysis^[^ ^14^, ^18^ ^]^ or for ex situ analysis after laborious extraction procedures.^[^ ^25^, ^26^ ^]^ Enzyme‐based electrochemical biosensors have been integrated in solid,^[^ ^14^, ^18^, ^27^, ^28^, ^29^ ^]^ hollow,^[^ ^30^, ^31^, ^32^, ^33^, ^34^ ^]^ or more recently, hydrogel MN devices.^[^ ^35^, ^36^ ^]^

Solid microneedles coated with metallic and biocatalytic layers are popular for electrochemical biosensing.^[^ ^18^, ^21^ ^]^ Coated MNs provide direct access to ISF for rapid analysis. Limitations include toxicity and stability concerns related to leaching, debris, and/or the direct contact of electrodes with internal tissues. Thin film metal electrodes are typically employed that have limited surface area and electrochemical potential window stability. Nanostructured Au and CNT coatings have been applied to solid MNs to enhance the electroactive surface area.^[^ ^28^, ^29^ ^]^ High surface area electrodes can enable higher biocatalyst loadings (e.g., enzyme and redox mediator) to increase the current output (sensor signal). The pore structure plays an important role in bioelectrocatalytic reactions; for example, mesopores can be desirable to achieve high enzyme loading, stabilization and electron transfer efficiency, while larger pores (e.g., macropores) favor faster mass transport.^[^ ^10^, ^13^ ^]^ Highly porous Au coatings with pores of ≈25 µm size provided well‐defined catalytic current signals and permitted CGM for 12 h on artificial skin.^[^ ^28^ ^]^ Tehrani et al. elegantly developed Pt‐coated MNs comprising an enzyme‐chitosan sensor layer with an external biocompatible synthetic polymer coating.^[^ ^14^ ^]^ The fabrication process was complex but scalable and permitted CGM for 12 h and 5 h on artificial and human skin, respectively. Freeman et al. recently developed a third generation lactate sensor that employed a porous carbon‐coated biosensor with a Ag/AgCl reference electrode in a multiarray MN design comprising 1 mm long needles.^[^ ^18^ ^]^ Continuous monitoring was demonstrated for 2.5 h on skin in human trials.^[^ ^18^ ^]^

Hollow microneedles are solid MNs comprising hollow channels that permit ISF extraction to electrodes positioned inside or at the back of the channels. The channels can offer physical protection to electrodes but are less effective for skin penetration compared to solid MNs. The hollow cavities are also prone to blockages. The access to ISF is less direct compared to solid MNs, resulting in longer activation (“pre‐heating”) and body‐to‐sensor lag times.^[^ ^37^ ^]^ Mohan et al. reported an alcohol biosensor comprising a metal wire inserted into a MN channel that was subsequently modified with alcohol oxidase, chitosan, and the fluoropolymer, Nafion.^[^ ^38^ ^]^ Biosensing was demonstrated in artificial skin but monitoring beyond 1 min was not reported.^[^ ^38^ ^]^ Windmiller et al. and later Teymourian et al. packed biocatalytic carbon paste electrodes in hollow MNs for lactate and glucose sensing, respectively.^[^ ^31^, ^34^ ^]^ Synthetic polyvinyl chloride membranes were used to facilitate CGM for ≈6 h in artificial ISF.^[^ ^34^ ^]^ Parrilla et al. reported a versatile MN biosensor compatible with screen‐printed electrodes.^[^ ^30^ ^]^ The Nafion‐coated bioelectrode was taped to the back of 1 mm long synthetic polymer MNs to achieve CGM in buffer for 7.5 h with a mass transport‐enhancing syringe pump.^[^ ^30^ ^]^

Hydrogel or “hydrogel swelling” microneedles are the latest type of MNs to emerge for transdermal (in situ) or ex situ biosensing.^[^ ^25^, ^35^, ^36^, ^39^ ^]^ In the dry state, the hydrogel MNs should provide sufficient mechanical strength to penetrate the skin with minimal force. After penetration, the polymer hydrogels adsorb ISF via osmotic forces through their porous hydrophilic networks. Hydrogel MNs are typically fabricated using synthetic polymers such as Gantrez and polyethylene glycols (PEGs) via low‐cost and scalable micromolding.^[^ ^36^, ^40^ ^]^ Polysaccharides and their derivatives offer attractive properties including exceptional swellability, biocompatibility, sustainability, and biodegradability.^[^ ^41^, ^42^ ^]^ On the other hand, polysaccharides generally exhibit limited mechanical strength and chemical resistance. For example, hydrogel MNs based on hyaluronic acid, without crosslinking, dissolved completely within 10 min in mouse skin ISF.^[^ ^43^ ^]^ The first hydrogel MN biosensors, an important inspiration for this work, exploited the swellable properties of hydrophilic crosslinked MNs to extract ISF for ex situ analysis after biomarker extraction from the MNs.^[^ ^25^, ^44^ ^]^

To the best of our knowledge, there are only two reports where an enzyme‐based electrochemical sensor has been combined with hydrogel MNs for transdermal biomarker detection and/or monitoring.^[^ ^35^, ^36^ ^]^ Caliò et al. reported a crosslinked PEG‐diacrylate/enzyme‐based MN array prepared using multi‐step lithography.^[^ ^36^ ^]^ The biosensor electrode was integrated via thin film gold deposition directly on the hydrogel, apparently without an adhesion layer to stabilize the electrode layer. The sensor permitted glucose detection up to 4 mmol L^−1^ levels, below the requirements for practical glucose sensing where blood glucose levels of diabetic patients can be 30 mmol L^−1^.^[^ ^2^ ^]^ The sensor appeared to be prone to electrode delamination, enzyme leaching, and risks associated with cytotoxicity via leaching and/or direct contact of Fc and enzyme with internal skin tissues. More recently, Zheng et al. reported micromolded UV‐crosslinked hyaluronic acid‐methacrylate MNs containing maltose as an “osmolyte” to drive ISF extraction from mouse skin.^[^ ^35^ ^]^ The bioelectrode was positioned at the back of the device without an apparent robust integration process. Detection over a limited range up to a glucose concentration of 8–16 mmol L^−1^ was demonstrated. Continuous monitoring was not reported. Further limitations include the risk of osmolyte leaching and the limited storage stability of the hygroscopic MNs. Considering the report of Park et al., dry‐state UV‐crosslinked hyaluronic acid MNs that dissolve rapidly are insufficiently robust for longer term continuous transdermal monitoring over several weeks.^[^ ^45^ ^]^

**Figure 1:**
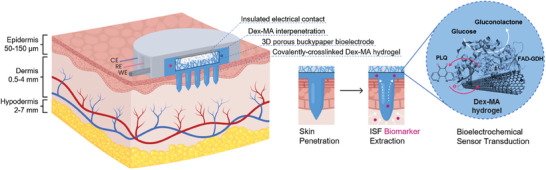
The integrated biopolymer hydrogel‐bioelectroenzymatic sensor concept that involves skin penetration then transdermal ISF extraction and glucose transport from the dermis layer to a three‐electrode bioelectrocatalytic sensor. Sensor transduction relies on mediated bioelectrocatalytic glucose oxidation at a buckypaper‐based bioelectrode (working electrode, WE) with an immobilized FAD‐GDH enzyme and an orthoquinone redox mediator (PLQ) positioned within a covalent chemically crosslinked Dex‐MA hydrogel matrix. The concept benefits from polymer interpenetration into the 3D porous buckypaper bioelectrode.

In this study, we extend the recent use of synthetic polymers and degradable biopolymer hydrogels to a strategy that focusses on the fabrication of highly crosslinked polysaccharide MNs with restricted swellability and high resistance. To the best of our knowledge, no hydrogel MN array has been developed that enables transdermal enzyme‐based biosensing beyond a few minutes, due to limitations in the materials and electrodes employed. We move away from the use of complex fabrication methods and synthetic polymers to instead focus on simple and ecofriendly processes with biosourced materials. The transdermal hydrogel microneedle‐bioelectroenzymatic sensing concept explored here relies on the skin application of a microneedle array that (i) penetrates the skin, (ii) enables dermal ISF extraction to the integrated electrodes via osmosis, and (iii) permits second generation enzyme‐based electrochemical detection and continuous monitoring of glucose (and eventually other biomarkers) in the ISF (**Figure** **1**). The swellable hydrogel microneedles were constructed using chemically crosslinked dextran‐methacrylate (Dex‐MA). Buckypaper was chosen as the electrode material to construct the biosensor working electrode (WE) due to properties such as its high surface area and high conductivity, as well as attractive properties for wearable devices such as low thickness (e.g., 5–300 µm) and weight, and some bendability. Buckypapers are self‐supporting sheets of randomly entangled carbon nanotubes with a 3D porous nanostructure that is conducive for the immobilization and electrical contact of enzymes and guest molecules for bioelectrocatalytic reactions.^[^ ^9^ ^]^ Here, the paper‐like buckypaper electrode was coated with phenanthroline quinone (PLQ) and fungal FAD‐GDH, and employed as the working electrode (WE, BP_PLQ_‐GDH). Phenanthroline quinone is an electron transfer mediator that enables electron transfer between the electrode and the enzyme for second generation bioelectrocatalytic glucose sensing. Fungal FAD‐GDH is now widely employed in commercial glucose test strips for glucose oxidation, but to the best of our knowledge, not yet used for continuous glucose monitoring devices. Unlike GOx, FAD‐GDH is O_2_ insensitive and could therefore be employed to offer higher bioelectrocatalytic efficiency for second generation CGM devices.

The microneedle‐electrochemical biosensor array is envisaged here for transdermal glucose monitoring, but could be expanded for the monitoring of alternative biomarkers via the simple insertion of alternative bioelectrodes. Interestingly, this approach opens up the possibility to integrate reference (RE) and counter (CE) electrodes in the same microneedle array to achieve a convenient and practical fully integrated (e.g., three‐electrode) monitoring device.

## 2 Results

### 2.1 Synthesis and Characterization of Crosslinked Dex‐MA Hydrogel MNs

**Figure 2:**
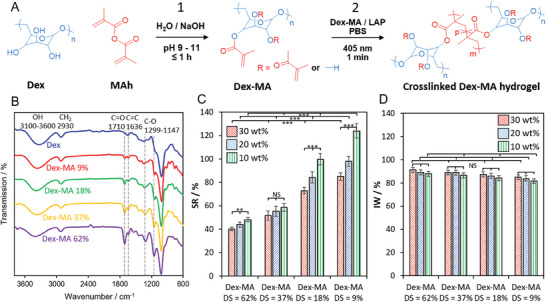
A) Reaction scheme for (1) aqueous Dex‐MA synthesis and (2) visible light‐induced chemical crosslinking of Dex‐MA to obtain covalently crosslinked Dex‐MA hydrogels (DS = 9–62%). B) FTIR spectra of Dex and Dex‐MA polymers. C) Swelling ratio (SR) and D) insoluble weight fraction (IW) at equilibrium for crosslinked Dex‐MA materials prepared with 10–30 wt% polymer in 0.01 mol L^−1^ PBS pH 7.4 with 1% LAP. SR and IW data is presented as mean ± SD (*n =* 3). Statistical significance was calculated using one‐way ANOVA; NS, ≥ 0.05, ^**^ *p* < 0.01 and ^***^ *p* < 0.005.

A series of photocrosslinkable methacrylate‐modified dextran polymers (Dex‐MA) were prepared with different degrees of substitution (DS = 9–62%) via an aqueous synthesis protocol. Until now, to the best of our knowledge, dextran methacrylation required the use of toxic organic solvents, reagents and/or organic catalysts, inert gas, and reaction times of > 10 h.^[^ ^42^, ^46^, ^47^ ^]^ The reaction was achieved here using small amounts of methacrylic anhydride (MAh) at pH 9–11 in air for ≤ 1 h (**Figure** **2**A). The reaction proceeds via nucleophilic attack from the hydroxyl groups of dextran (Dex) at the carbonyl groups of MAh, with methacrylic acid formed as the byproduct. Polymers with different DS were obtained using different number equivalents of MAh (0.0625–0.5 eq.). The FTIR spectra of Dex and Dex‐MA confirmed the presence of the characteristic ─OH and ─CH_2_ groups as well as the emergence of ester bonds due to incorporated methacrylate groups (Figure 1B). ^1^H NMR was used to determine the DS values and confirm the formation of high purity Dex‐MA (Figure S1, Supporting Information). The reaction was very efficient compared to existing protocols. For example, yields of 85–90% after ≤ 1 h of reaction were obtained in contrast to yields of 70–90% after 48 h of reaction in organic solvent under a nitrogen atmosphere.^[^ ^46^ ^]^ A basic pH was maintained to ensure the high reactivity of the hydroxyl groups.

Chemical crosslinking was achieved by light irradiation for 1 min at λ = 405 nm with polymer formulations containing 1 wt% phenyl‐2,4,6‐trimethylbenzoylphosphinate (LAP) as the photoinitiator in 0.01 mol L^−1^ phosphate buffer saline (PBS) pH 7.4 (Figure 2A). LAP offers improved water solubility, cytocompatibility and visible light reactivity compared to common Irgacure derivatives.^[^ ^48^, ^49^ ^]^ Crosslinking was performed in the solid state (“dry‐state”) as opposed to the more conventional crosslinking of hydrogels in solution (“wet‐state”).^[^ ^25^, ^35^, ^40^, ^42^ ^]^ In this study, “dry‐state” refers to hydrogel materials that were dried at room temperature for 24 h to remove water. In contrast “wet‐state” refers to equilibrium buffer swollen hydrogel materials. This strategy was chosen to minimize material shrinkage to facilitate the fabrication of complex microneedle structures using highly swellable materials.^[^ ^41^, ^45^ ^]^

The crosslinked Dex‐MA materials exhibited equilibrium swellability ratios (SRs) ranging from 40.2 ± 1.5% to 124 ± 6.1% in 0.01 mol L^−1^ PBS pH 7.4, depending on the polymer DS and mass fraction employed (Figure 2C). The decrease in swellability with increasing polymer DS is consistent with an increase in the crosslinking density of the material. The increased crosslinked density is enabled by the higher number of methacrylate groups present on the polymer. The decrease in swellability with an increasing amount of polymer is consistent with an increased crosslinking density due to the high density of methacrylate groups in the more concentrated formulation. The SRs are high compared to crosslinked PEG‐based materials and very low compared to methacrylate‐crosslinked hyaluronic acid and dextran hydrogels that exhibited “super‐swelling” SRs in the range of 200–900% or 5000%.^[^ ^42^, ^45^ ^]^ The significantly lower SRs obtained here underline a particularly high efficiency of the dry‐state Dex‐MA crosslinking reaction. The obtained Dex‐MA materials benefitted from a high density of crosslinks with intimate intertwining and stacking interactions between polymers in the solid state.^[^ ^42^ ^]^

The stability of the crosslinked Dex‐MA hydrogels was evaluated at room temperature in 0.01 mol L^−1^ PBS pH 7.4 (Figure 2D). The hydrogel weight loss corresponds to leakage of uncrosslinked Dex‐MA, unreacted LAP and its byproducts, and chemical degradation, e.g., via hydrolysis of methacrylate ester bonds.^[^ ^50^ ^]^ The unmodified native dextran completely dissolved in a few minutes. In contrast, high insoluble weight fractions of 78–92 ± 2% were observed after 7 days for Dex‐MA hydrogels, consistent with robust crosslinked polysaccharide materials, for example, compared to previously described methacrylated dextran and hyaluronic acid‐based hydrogels.^[^ ^42^, ^45^ ^]^ In the first and only report of Dex‐MA MNs, to our knowledge, crosslinked Dex‐MA hydrogels (DS = 20%) exhibited IWs of ≈ 66–88% after 36 h in 0.01 mol L^−1^ PBS.^[^ ^42^ ^]^

The mechanical properties of dry crosslinked Dex‐MA hydrogels were determined by compression tests to investigate their skin perforation ability. Stress–strain curves were recorded for the crosslinked materials obtained from formulations prepared with different polymer DS (9–62%) and mass fraction (10–30 wt%). Figure S2 (Supporting Information) shows representative stress–strain curves obtained for dry Dex‐MA hydrogels prepared with DS = 9% and DS = 37%. The dry crosslinked Dex‐MA and Dex materials exhibited compression modulus values ranging from ≈35 to 330–340 MPa that increased as the polymer mass fraction increased from 10 to 30 wt% (Table S1, Supporting Information). These values are higher than the ultimate tensile strength values 0.1–28 MPa reported for human and animal skin samples, and therefore promising with respect to the envisaged skin penetration application.^[^ ^51^, ^52^ ^]^ The polymer DS had no significant effect on the compression moduli while Dex exhibited similar compression moduli to the crosslinked Dex‐MA materials. The mechanical properties of the dry crosslinked Dex‐MA materials are therefore governed by the intrinsic interactions and entanglement between dextran chains as opposed to the inter‐chain covalent crosslinks.

### 2.2 Fabrication of Crosslinked Dex‐MA Hydrogel MNs and Their Characterization in Solution and Artificial Skin

**Figure 3:**
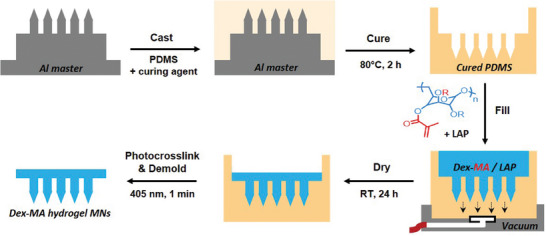
Microfabrication scheme to obtain Dex‐MA MNs via PDMS micromolding with a vacuum manifold and dry‐state visible light photocrosslinking.

Dex‐MA microneedle arrays were fabricated via a simple and convenient micromolding procedure with dry‐state visible light crosslinking, as illustrated in **Figure** **3**. Briefly, Dex‐MA formulations prepared in 0.01 mol L^−1^ PBS pH 7.4 with 20 wt% polymer and 1 wt% LAP were cast into PDMS molds (Figure S3, Supporting Information) obtained from Al master templates (Figure S4A, Supporting Information).

**Figure 4:**
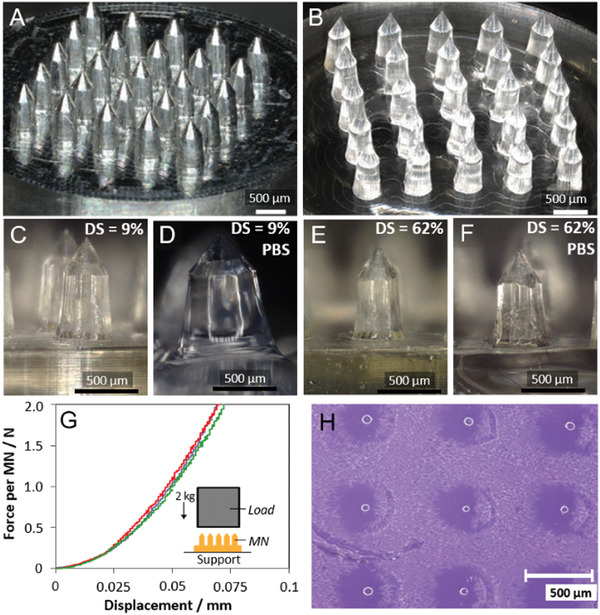
A–F) Digital images for (A) Al master and (B) dry Dex‐MA hydrogel MNs (DS = 9%). C,D) Dex‐MA MNs (DS = 9%) and (E, F) Dex‐MA MNs (DS = 62%) before and after equilibrium swelling in 0.01 mol L^−1^ PBS pH 7.4. G) Force–displacement curves obtained for MNs (DS = 37%): design A (purple), B (red), and C (green), with (inset) experimental set‐up sketch. H) Digital image of perforated artificial skin model (hard skin: 24 wt% gelatin: 1 wt% agar) after application of Dex‐MA hydrogel MNs (DS = 9%).

A vacuum manifold was used to effectively fill the microcavities of the PDMS mold with the polymer formulation with minimal air bubbles. After drying at room temperature in air, the polymer formulation was crosslinked in the dry‐state in the PDMS mold for 1 min at λ = 405 nm (75 mW cm^−2^), then the Dex‐MA MN array was obtained after demolding. The various dimensions of the Al MN master templates are illustrated and summarized in Figure S4 and Table S2 (Supporting Information), respectively. A high resolution digital image of the Al master (design A) with 790 ± 3 µm MN heights is shown in **Figure** **4**A. Images of the various Al master designs used in this work are shown in Figure S5 (Supporting Information). Unless stated otherwise, all MN data reported herein was obtained using the Al master design A with 790 ± 3 µm needle heights. Furthermore, unless stated otherwise, all hydrogel MNs were obtained using formulations containing 20 wt% polymer and 1 wt% LAP in 0.01 mol L^−1^ PBS pH 7.4.

The micromolding with dry‐state crosslinking process permitted the convenient fabrication of crosslinked Dex‐MA hydrogel MN arrays with well‐defined “pencil tip” needle structures from polymers with DS = 9–62%. Figure 4B shows a representative example of a Dex‐MA (DS = 9%) MN array with microneedle lengths of 783 ± 4 (design A). Images of MN arrays prepared with different dimensions, and a summary of the experimentally determined critical dimensions for various MN arrays, are available in Figure S6 and Table S3 (Supporting Information), respectively. For all MN arrays, 6 of the 7 critical dimensions varied by less than 4% compared to those of the corresponding Al master template. All 7 dimensions exhibited a repeatability of < 1 RSD% (*n =* 3). The needle tip diameter, the exception to the rule, was consistently 19–22% smaller compared to the theoretical dimension based on the Al master. The reduction in tip size is beneficial since sharper structures require less force for penetration. The unexpected “pencil sharpening” effect may be due to localized shrinkage effects due to ineffective solvent removal at the tips during the precrosslinking drying step, but this was not studied further. By comparison, wet‐state crosslinked Dex‐MA MNs suffered from major issues with material shrinkage and cracking (Figure S7, Supporting Information) hence the use of dry‐state crosslinking proved critical.

Digital images recorded at individual MNs before after PBS swelling to equilibrium revealed the impact of swelling on the microstructure of the MNs (Figure 4C vs 4D and Figure 4E vs 4F). For the high swellability Dex‐MA MNs (DS = 9%) with SR = 95%, the dimensions expand significantly by up to ≈100% (Figure 4C,D). In stark contrast, for the highly crosslinked MNs (DS = 62%) with SR = 42%, the dimensions expanded by a maximum of only ≤ 1%. The swelling expansion data for the Dex‐MA MN arrays, prepared with different designs, is shown in Table S4 (Supporting Information). The dimension expansions after swelling in 0.01 mol L^−1^ PBS pH 7.4 are different at different points on the fabricated microstructures. This is consistent with anisotropy in the crosslinked hydrogel, for example, due to a gradient of crosslinking densities.

The skin penetration performance of dry MN arrays was evaluated by compression testing and simple perforation tests. Figure 4G shows the force–displacement plot obtained for MN arrays (DS = 37%, 20 wt%) subjected to a force up to 50 N against a flat metal support. The test is demanding, considering the average compression force exerted by a user of a MN device on soft matter (skin) is evaluated to be ≈8 N.^[^ ^14^ ^]^ A short‐lived quasi‐linear displacement curve was observed at low compressive force that transitioned to a stable linear slope (≈45 N mm^−1^) at a force of > 2 N for the three different MN designs. Digital images obtained after the compressive test revealed some flattening of the MN tips (Figure S8, Supporting Information). A maximum loss in needle height of 8.9 ± 1.6% was observed (Table S5, Supporting Information). No further evidence of material fracture or breakage was observed.

The capacity of the MNs to penetrate skin with an applied vertical load of 2 kg was qualitatively evaluated using an artificial “hard” skin model comprising 24 wt% gelatin, 1 wt% agar, and 75% 0.01 mol L^−1^ PBS pH 7.4. Note: details regarding the development and optimization of the “hard” and “soft” skin models are available in Figure S9 and Table S6 (Supporting Information). The artificial skin models comprising PBS or ISF were used here as simple but convenient mimics of the dermis to demonstrate skin penetration and fluid swelling before future validation on an animal/human model.^[^ ^53^ ^]^ Skin models prepared with 0.01 mol L^−1^ PBS pH 7.4 rather than artificial ISF were employed for skin penetration tests due to their optical transparency. Figure 4H and Figure S10 (Supporting Information) show examples of the penetration profiles that remain in the skin model after application and removal of the MN device. The images obtained for MNs prepared with different designs display the general expected penetration profiles. 3D laser scanning microscopy of a penetration profile obtained from a Dex‐MA (DS = 9%, 20 wt%) hydrogel MN array provided experimental evidence for qualitatively homogeneous skin penetration (Figure S11, Supporting Information).

### 2.3 Characterization and Second Generation Bioelectrocatalytic Glucose Sensing with a Nanostructured Buckypaper Biosensor in Buffer and Artificial ISF

Self‐supporting carbon nanotube buckypaper bioelectrodes have emerged as attractive porous electrodes for wearable bioelectrocatalytic sensors owing to their large specific surface area and high electrical conductivity, flexibility, biocompatibility, and thin form factor (≈200 µm thick).^[^ ^7^, ^8^, ^9^ ^]^ Here, we employed a lab‐made carbon nanotube buckypaper bioelectrode as the biosensor that was prepared via vacuum filtration fabrication and drop‐coating modification protocols. After surface modification, an electrical wire was attached to the back of the buckypaper electrode via carbon paste to permit the electrical connection of the electrode to the potentiostat. The carbon paste and conducting wire contact were subsequently coated with an insulating silicone paste layer. We employed a bioelectrode architecture that employed an orthoquinone as the redox mediator, phenanthroline quinone, and fungal FAD‐glucose dehydrogenase. This type of bioelectrode, herein referred to as BP_PLQ_‐GDH, was previously reported as a catalytically active but limited stability bioanode for biofuel cells.^[^ ^7^ ^]^ Here we explore the development and optimization of the buckypaper bioelectrode for low potential second generation glucose biosensing and CGM. In principle, a wide range of mediators and/or enzymes can be immobilized at buckypaper electrodes via the drop‐coating protocols, although this was not investigated here.

**Figure 5:**
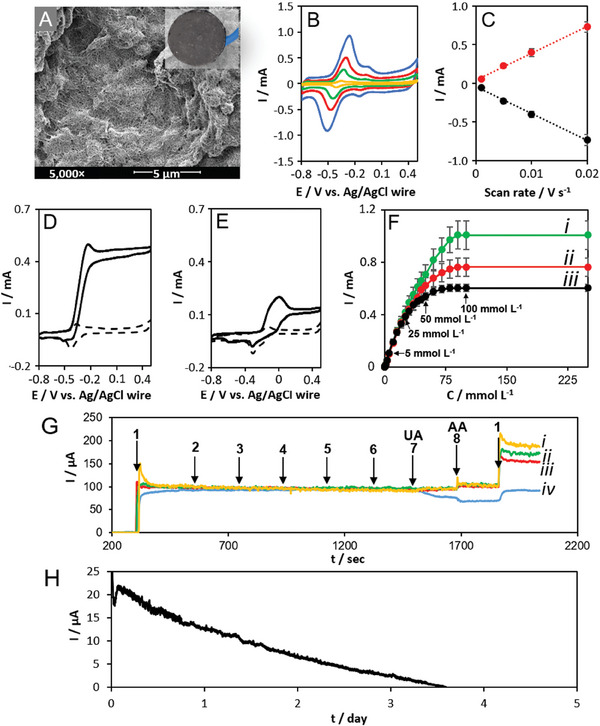
A) SEM image and (inset) digital photograph of the BP_PLQ_‐GDH electrode. B,C) CVs and corresponding peak current versus scan rate plot for BP_PLQ_‐GDH in 0.1 mol L^−1^ PB pH 7.4 at varying scan rates: 1 mV s^−1^ (red), 5 mV s^−1^ (green), 10 mV s^−1^ (red), and 20 mV s^−1^ (blue). The peak current versus scan rate plot data is presented as mean ± SD (*n =* 3). D,E) CVs of BP_PLQ_‐GDH in 0.1 mol L^−1^ PB and artificial ISF at pH 7.4, respectively, recorded at 1 mV s^−1^ in the absence (dash) and presence (solid) of 0.1 mol L^−1^ glucose. F) Glucose oxidation current versus concentration plotted from fixed potential CA recorded at BP_PLQ_‐GDH in 0.1 mol L^−1^ PB pH 7.4 at *E*_app_ = 0.1 V (i, green), −0.1 V (ii, red), and −0.3 V (iii, black) versus Ag/AgCl at 500 rpm. The CA current versus concentration plot data is presented as mean ± SD (*n =* 3). G) Fixed potential CAs recorded at BP_PLQ_‐GDH at *E*_app_ = 0 V (i, yellow), −0.1 V (ii, green), −0.2 V (iii, red), and −0.3 V (iv, blue) versus Ag/AgCl in 0.1 mol L^−1^ PB pH 7.4 at 500 rpm with sequential addition of 1) 5 mmol L^−1^ glucose, 2) 0.2 mmol L^−1^ acetaminophen, 3) 1 mmol L^−1^ cholesterol, 4) 8 mmol L^−1^ urea, 5) 2 mmol L^−1^ lactate, 6) 0.3 mmol L^−1^ galactose, 7) 0.5 mmol L^−1^ uric acid, 8) 0.1 mol L^−1^ ascorbic acid, then a repeat addition of 1). H) CGM by CA in artificial ISF with 5 mmol L^−1^ glucose at 0 V versus Ag/AgCl.

**Figure** **5**A shows the randomly entangled and porous nanostructured morphology of the buckypaper bioelectrode comprising multiwalled carbon nanotubes. The presence and electrochemical accessibility of the redox mediator was evaluated by recording cyclic voltammograms (CVs) in 0.1 mol L^−1^ phosphate buffer (PB) pH 7.4 at BP_PLQ_‐GDH (Figure 5B) and BP_PLQ_ (Figure S12, Supporting Information) electrodes in the presence and absence of immobilized enzyme, respectively. The o‐quinone/o‐hydroquinone redox mediator at the 6 mm diameter electrodes gave well‐defined responses at different scan rates in the absence and presence of the enzyme. The closely spaced redox couples are considered to be independent 2‐electron redox processes due to the neutral pH and buffer content.^[^ ^54^ ^]^ The linear Randles–Ševčík plots of peak current versus scan rate confirm the surface‐bound nature of the physically adsorbed quinone mediator (Figure 5C; Figure S12, Supporting Information). The peak‐to‐peak separation values (Δ*E*_p_) of ≤ 85 ± 5 mV at 1 mV s^−1^ at BP_PLQ_‐GDH are consistent with attractive apparent electron transfer kinetics. The electrochemical parameters such as the half‐wave potential (*E*_1/2_), Δ*E*_p_, background capacitance (*Q*), and mediator surface coverage (Γ_PLQ_) determined in simple and complex buffers are summarized in Table s7 (Supporting Information).

After enzyme immobilization, a threefold decrease in the background capacitance, an increase in the Δ*E*_p_, and no significant change in Γ_PLQ_ was observed for the comparative experiment performed in 0.1 mol L^−1^ PB (BP_PLQ_ versus BP_PLQ_‐GDH). The insulating glycosylated protein shell of the enzyme therefore partially blocked charge transfer at the bioelectrode interface. In more challenging chloride‐containing artificial ISF and 0.01 mol L^−1^ PBS, the *E*_1/2_ values for the quinone couple shifted by ≈ +0.08 V while the surface concentration decreased twofold compared to the values obtained in 0.1 mol L^−1^ PB. The significant diminution in the surface coverage appears to be related to solvent/electrolyte interactions that, for example, provoked leaching, but this was not studied further. The potential shift originated from the chlorinated Ag wire pseudo‐reference electrode whose potential varied with the Cl^−^ concentration. Note: further discussion and experimental data to validate the pseudo‐reference electrode shifts are available in Figure S13 and Table S8 (Supporting Information).

The bioelectrocatalytic glucose oxidation response in simple buffer solution and complex artificial ISF at pH 7.4 was subsequently explored toward the transdermal biosensing application. Well‐defined sigmoidal‐type voltammograms were observed at BP_PLQ_‐GDH at 1 mV s^−1^ in both 0.1 mol L^−1^ PB and artificial ISF in the presence of a high (saturating) glucose concentration, as shown in Figure 5D,E, respectively. Steady–state catalytic currents of 1.68 ± 0.06 and 0.46 ± 0.04 mA cm^−2^ were obtained in 0.1 mol L^−1^ PB and artificial ISF, respectively. The important loss in catalytic output is consistent with the presence of additional inhibiting, deactivating, and/or fouling species in the complex ISF medium. The catalytic current outputs nevertheless remain large (≈0.45 mA cm^−2^) and reliable due to the efficient electrical wiring of the enzyme and the high surface area and conductivity of the buckypaper electrode (Table s9, Supporting Information). Similar catalytic currents were obtained in the absence of oxygen (Figure S14 and Table s9, Supporting Information), consistent with the well‐known oxygen insensitivity of this fungal dehydrogenase.

Amperometric glucose sensing was initially explored at fixed potentials of *E*_app_ = −0.3, −0.1, and 0.1 V versus Ag/AgCl under hydrodynamic conditions (500 revolutions per minute, rpm). We targeted the development of a glucose biosensor capable of operating at low potentials to minimize electro‐oxidative interferences. Figure 5F shows how the steady–state catalytic current increased with increasing glucose concentration until a classical glucose‐saturating plateau was reached at high concentrations. The saturation current occurred at a glucose concentration of ≈0.1 mol L^−1^ for the three potentials explored. The corresponding fixed potential chronoamperograms (CAs) used to determine the catalytic currents are available in Figure S15 (Supporting Information). The potential has a negligible effect on the current output at low glucose concentrations. At high concentrations of ≥ 25 mmol L^−1^, higher catalytic currents were achieved with increasing applied potential due to the increasing driving force and consequent reaction efficiency. The improved reaction is explained by a more effective electrochemical re‐oxidation of the reduced quinone at high substrate concentrations. The results highlight an important benefit of using higher applied potentials; for example, the possibility to expand the upper limit of the linear dynamic range from 25 mmol L^−1^ (*E*_app_ = −0.3 V) to 35 mmol L^−1^ (*E*_app_ = 0.1 V) versus Ag/AgCl. The sensitivity, limit of detection and response time were relatively independent of the applied potential (Table S10, Supporting Information). The ability to increase the upper concentration limit of the biosensor is important since the blood glucose concentration in diabetic patients can be as high as 30 mmol L^−1^.^[^ ^2^ ^]^ It is noted that blood glucose levels are generally in the range of 4–8 mmol L^−1^.^[^ ^2^ ^]^

Under quiescent conditions, smaller bioelectrocatalytic currents and a decrease in sensitivity were observed compared to the experiments performed under hydrodynamic conditions. The applied potential had a less significant impact under this “flux limited” substrate condition. Under quiescent conditions, the glucose flux was reduced and the dynamic sensor range became linear up to 50 mmol L^−1^ (Figure S16, Supporting Information). The biosensor performance characteristics determined under quiescent conditions are also summarized in Table S10 (Supporting Information).

The selectivity of the BP_PLQ_‐GDH biosensor was evaluated in artificial ISF containing physiologically relevant 5 mmol L^−1^ glucose. For these experiments, glucose was first added, followed by the incremental addition of 7 potentially interfering species at physiologically relevant ISF concentrations, followed by a final 5 mmol L^−1^ glucose addition. Figure 5G shows the amperometric responses obtained at 4 different fixed potentials in the range of −0.3 to 0 V versus Ag/AgCl. Fixed potentials of −0.2 or −0.1 V versus Ag/AgCl are preferred for minimizing the combined electrochemical interference of ascorbic acid (AA, ascorbate) and uric acid (UA, urate) to < 10% of the initial glucose response (6.0% and 6.8%, respectively). An interference level of 10% is generally considered acceptable.^[^ ^2^ ^]^ For example, at −0.1 V, the UA interference is negligible while AA oxidation provokes the 6.8% interference level. For the more extreme applied potentials of 0 and −0.3 V, the combined interference level was unacceptable at ≈25% (Table S11, Supporting Information). At −0.3 V, for example, the glucose response is strongly affected by the electrochemical reduction of both UA and AA, while for the more positive potentials, the principle electrochemical interference was the oxidation of ascorbic acid. Note: supplementary references relating to the composition of the ISF interferents are cited in Supporting Information. At the end of each 30 min experiment, a second addition of 5 mmol L^−1^ glucose was made to evaluate the glucose sensor response. After the second glucose addition, a catalytic glucose oxidation current was again observed but with notable catalytic current losses. For *E*_app_ = 0 and −0.3 V, the glucose current response was 91.5% and 24.5% that of the initial glucose addition, respectively (Table S11, Supporting Information), hence there is an important trade‐off between driving force and minimizing electro‐active interferences in ISF media. The notable catalytic current losses can be attributed to numerous factors relating to the 8 added potential interferences, ranging from steric affects, e.g., biofouling, that can impede glucose diffusion to/from the enzyme or electro‐oxidation of PLQ, to side reactions or local pH changes that affect the reactivity of PLQ and/or the enzyme. Based on these studies, a fixed potential of −0.1 V in artificial ISF versus Ag/AgCl pseudo‐reference was chosen as a compromise to maximize the transduction signal while minimizing electroactive interferences and other side reactions resulting from the presence of the interfering species.

The CGM performance of the buckypaper bioelectrode was subsequently evaluated in artificial ISF with 5 mmol L^−1^ glucose at the optimized applied potential of −0.1 V versus Ag/AgCl wire (Figure 5H). The biosensor exhibited a respectable monitoring performance that lasted 3.5 ± 0.3 days. The CGM performance is promising for a GDH‐based electrode operating in artificial ISF, considering that the bioelectrode has no protecting and/or diffusion‐limiting polymer membrane. For comparison, Tsujimura et al. demonstrated CGM for 3 days with a GDH‐modified Os‐based redox‐hydrogel bioelectrode operating in simple PBS.^[^ ^12^ ^]^

### 2.4 Dex‐MA Microneedle‐Electrochemical Glucose Biosensor Arrays

**Figure 6:**
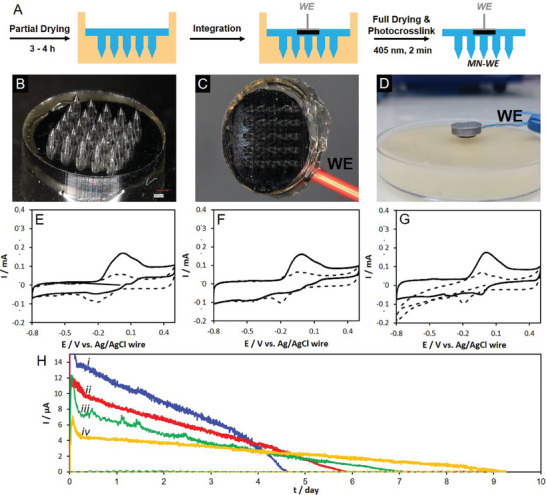
A) Electrode integration scheme to obtain Dex‐MA hydrogel MNs with a single integrated BP_PLQ_‐GDH bioelectrode (MN‐WE). B,C) Digital microscopy images of the MN‐WE device, and D) digital photograph of an applied MN sensor device on artificial skin. E–G) CVs recorded at 1 mV s^−1^ at MN‐WE (DS = 37%) with external Ag/AgCl and Pt wire counter electrodes in 0.01 mol L^−1^PBS, artificial ISF, and artificial ISF‐containing soft skin (4 wt% gelatin with 1 wt% agar), respectively, in the absence (dash) and presence (solid) of 0.1 mol L^−1^ glucose. H) Fixed potential CGM recorded for MN‐WE in artificial ISF with 5 mmol L^−1^ glucose for devices prepared with different polymer DS: 9% (i, blue), 18% (ii, red), 37% (iii, green), and 62% (iv, yellow) at 0 V versus Ag/AgCl.

**Figure 7:**
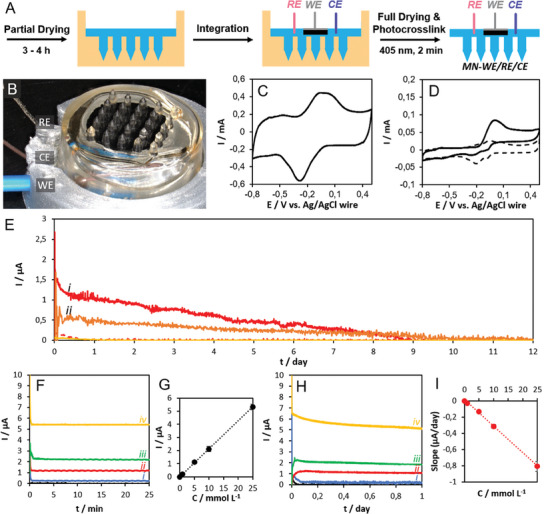
A) Electrode integration scheme to obtain Dex‐MA hydrogel MNs (DS = 62%) with a fully integrated three‐electrode biosensor (MN‐WE/RE/CE). B) Digital photograph of the fully integrated MN‐WE/RE/CE with 3D‐printed support. C) CV recorded at 20 mV s^−1^ in artificial ISF‐containing soft skin with MN‐WE/RE/CE in the absence of glucose. D) CVs recorded at 1 mV s^−1^ in soft skin with MN‐WE/RE/CE in the absence (dash) and presence (solid) of 0.1 mol L^−1^ glucose. E) Fixed potential CGM recorded at 0 V versus Ag/AgCl for MN‐WE/RE/CE in soft (i, red) and hard (ii, orange) skin models in (dash) the absence and (solid) presence of 5 mmol L^−1^ glucose under a force of 2 kg. F,H) Fixed potential CAs at *E*_app_ = 0 V versus Ag/AgCl in the absence (black line) and presence of different concentrations of glucose: (i, blue) 1 mmol L^−1^, (ii, red) 5 mmol L^−1^, (iii, green) 10 mmol L^−1^, and (iv, yellow) 20 mmol L^−1^) and the G,I) corresponding calibration curves obtained for MN‐WE/RE/CE in artificial ISF‐containing soft skin: F,G) periodic raw current calibration method and H,I) linear decay slope calibration. The current versus concentration calibration curve data is presented as mean ± SD (*n =* 3).

A “dual‐state” electrode integration and MN crosslinking process was developed that permitted the robust encapsulation of a single (**Figure** **6**A) or multiple (**Figure** **7**A) electrodes in a single MN array. The term “dual‐state” refers to the use of (i) wet‐state electrode integration, and (ii) dry‐state photocrosslinking. The dual‐state process relies on the simple insertion of prefabricated electrodes in a slightly more viscous Dex‐MA polymer formulation than used previously to obtain Dex‐MA MNs (Section 2.2.). To facilitate the positioning of the electrode(s) in the MN device, a partial drying step was employed to increase the viscosity of the polymer formulation. After complete drying then visible light crosslinking, the electrodes became fixed in the crosslinked Dex‐MA matrix of the MNs. After demolding and flipping of the integrated MN device, an additional 1 min irradiation step was performed vis‐à‐vis the needle tips (perpendicular to the MN base plate). The second irradiation step was employed to ensure that the needle structures were exposed to irradiation for at least 1 min without hindrance due to the presence of opaque electrodes in the MNs.

#### 2.4.1 Single Bioelectrode (MN‐WE) Devices for Transdermal Detection in the Artificial Soft Skin Model (4 wt% Gelatin with 1 wt% Agar)

Initial characterization studies focused on single bioelectrode MN devices (MN‐WE) for transdermal sensing with external Ag/AgCl pseudo‐reference and Pt wire counter electrodes. Figure 6B,C shows photographs of a typical MN‐WE device. High resolution digital images of MN‐WE devices prepared with crosslinked Dex‐MA (DS = 62%, 20 wt%) are shown in Figure S17 (Supporting Information). The MN‐WE devices prepared with 20 wt% formulations exhibited the same level of shape fidelity and repeatability as observed previously for Dex‐MA MNs prepared without an integrated WE (Section 2.2). The MN‐WE devices exhibited ≥ 96% correspondence to the Al master templates, except for the ≈20% narrower needle tips, and a repeatability of < 1.2%RSD for all measurements (Table S12, Supporting Information). The electrode integration process with the two‐step irradiation process had no notable impact on the fabrication of Dex‐MA MN arrays. Based on needle rupture experiments, the distance between the WE and the base of the MNs was roughly estimated as < 10 µm (Figure S18, Supporting Information). In this configuration, the number and dimensions of the needles employed would affect the extraction process (e.g., kinetics) and therefore preheating time, but not the volume of ISF analyzed.

The ability of MN‐WE devices to osmotically extract fluid and produce reliable electrochemical activity was initially confirmed by cyclic voltammetry in 0.1 mol L^−1^ PB at pH 7.4 (Figure S19, Supporting Information). The CVs recorded for MN‐WE devices prepared from Dex‐MA polymers with different DS are similar. The estimated electrochemical parameters, including the background capacitance and Γ_PLQ_, were similar for the MN devices, despite the different swellabilities and mechanical properties of the hydrogel matrices (Table S13, Supporting Information). Note: the peak potential shift and differences in terms of the closely spaced (overlapping) couples of the redox mediator compared to the nonintegrated bioelectrode (Figure 5B) occurred due to the presence of chloride in the swollen MNs. The chloride ions, present in the initial Dex‐MA formulation, were reconstituted during fluid uptake. The electroactivity recorded for MN‐WE in bulk 0.1 mol L^−1^ PB solution is therefore more representative of the behavior expected for CVs recorded in 0.01 mol L^−1^ PBS (Table S13, Supporting Information).

Electrochemical characterization of the MN‐WE devices in 0.01 mol L^−1^ PBS, artificial ISF and artificial ISF‐containing soft skin further confirmed the ability of the devices to extract fluid from more complex buffers and an ISF‐containing artificial skin model (Figure S20, Supporting Information). Figure 6D shows the set‐up for skin measurements. The extracted electrochemical parameters (Table S14, Supporting Information), highlight a roughly twofold increase in both the background capacitance and Γ_PLQ_ compared to the nonintegrated bioelectrode in both 0.01 mol L^−1^ PBS and artificial ISF. Together, these changes highlight, at least in part, an important enhancement in the electroactive surface area resulting from hydrogel interpenetration in the pore structure of the nanostructured buckypapers. The interpenetrated hydrogel permitted electrolyte permeation deeper into the 3D porous buckypaper electrode. While we consider an increase in the electroactive surface area to be the primary factor, other factors related to the electrolyte, charge transfer process, and surface chemistry also affect the surface capacitance and electroactive surface coverage.

The hydrogel interpenetration into the porous electrode structure could also help facilitate the mechanical fixation of the bioelectrode inside the hydrogel MNs. The Δ*E*p values are approximately two to threefold smaller for the integrated bioelectrodes compared to the nonintegrated bioelectrode. The remarkable enhancement in apparent heterogeneous electron transfer kinetics for the MN‐integrated bioelectrode may occur due to stabilizing intermolecular hydrogen bonding interactions between the orthoquinone mediator and dextran chains that facilitate electron mobility and proton transfer.^[^ ^54^ ^]^ It is noted that polymer interpenetration into the porous electrode occurs via diffusion during the integration process during which the bioelectrode is immersed in polymer solution for several hours.

The bioelectrocatalytic glucose oxidation activity of the MN‐WE bioelectrode devices was confirmed by cyclic voltammetry at 1 mV s^−1^ in 0.01 mol L^−1^ PBS, artificial ISF, and artificial ISF‐containing soft skin in the absence and presence of 0.1 mol L^−1^ glucose (Figure 6E–G). The recorded CVs are similar hence the globular protein (22 g L^−1^ of albumin) and the gelatinous skin exerted little effect on the bioelectrocatalytic reaction under these conditions. The steady–state bioelectrocatalytic currents of 0.24 ± 0.02 and 0.23 ± 0.3 mA cm^−2^ observed in 0.01 mol L^−1^ PBS and artificial ISF, respectively, were much lower than the 0.47 ± 0.3 and 0.46 ± 0.04 mA cm^−2^ observed under equivalent conditions for the nonintegrated bioelectrode prepared without Dex‐MA (Table S15, Supporting Information). The smaller catalytic currents are consistent with the hydrogel MN matrix acting as a glucose flux‐limiting membrane that reduces the local concentration of glucose at the bioelectrode interface. The effect of polymer DS on the bioelectrocatalytic activity of MN‐WE devices was also studied. Higher steady–state catalytic currents were observed for the more swellable Dex‐MA MNs prepared with lower polymer DS (Figure S21 and Table S15, Supporting Information). The catalytic current was increased by more than a factor of 2 by reducing the DS from 62% down to 9%. This revealing experiment highlights the possibility to modulate the glucose diffusion‐limiting behavior of the microneedles with careful control over the crosslinked polymer network.

Pushing toward the transdermal application, a series of crosslinked Dex‐MA hydrogel MN‐WE devices prepared with different polymer DS were evaluated for CGM in artificial ISF. Figure 6H shows the current–time response in the absence and presence of 5 mmol L^−1^ glucose at 0 V versus Ag/AgCl. The MN‐WE devices were capable of CGM in artificial ISF from 4.5 ± 0.3 days for MN‐WE (DS = 9%) up to 9.1 ± 0.3 days for MN‐WE (DS = 62%). The polymer DS therefore plays a crucial role on both the CGM lifetime and the catalytic current output. These results highlight the interest of employing a Dex‐MA polymer with (i) a low DS, for shorter duration CGM biosensing (≤4 days), and (ii) a high DS, for long‐term CGM (>5 days). Due to the beneficial Dex‐MA hydrogel matrix, all MN‐WE biosensors, irrespective of the polymer DS, outperformed the equivalent nonintegrated biosensor by at least 24 h in terms of CGM. In the best case, the CGM was increased by an average of 5.6 days that corresponds to a 160% increase in monitoring lifetime. In addition to the substrate flux‐limiting property, the covalently crosslinked hydrogel may also limit the flux and/or interaction of interfering and deactivating species with the immobilized enzyme, but this was not studied further. An important limitation of the MN‐WE devices concerns the stabilization or “pre‐heating” time, i.e., the time from the application of the device on the skin to achieving a stable response. The preheating time was 1.6 ± 0.1 h for the nonintegrated BP_PLQ_‐GDH bioelectrode in artificial ISF and ≈4.3 ± 0.5 h for the equivalent bioelectrode in the MN‐WE devices. The longer stabilization times reflect the osmotic extraction process and complexity associated with effective wetting and stabilization of the porous integrated electrode.

#### 2.4.2 Fully Integrated Bioelectrode (MN‐WE/RE/CE) Devices for Transdermal Detection in Soft and Hard Artificial Skin

Practical transdermal biosensors require a two or three‐electrode electrochemical cell comprising the working electrode (sensor) together with reference and counter electrodes. The RE provides a stable and reproducible ground potential while the CE completes the circuit. In the few published reports to date on transdermal electrochemical biosensing, the RE and CE electrodes were either (i) integrated on devices comprising independent MN arrays prepared with distinct materials and coatings,^[^ ^14^, ^18^ ^]^ (ii) inserted into the channels of distinct hollow MNs,^[^ ^34^, ^38^ ^]^ or (iii) attached at the back of the MN array.^[^ ^30^, ^35^, ^45^ ^]^

We simply extended the dual‐state WE integration process (Section 2.4.1) to permit the additional integration of CE and RE wire electrodes, as illustrated by the scheme in **Figure** **7**A. The polymer formulation with Dex‐MA (DS = 62%) at 20 wt% with 1% LAP was selected due to the attractive prospects for long‐term CGM, as observed in Section 2.4.1. For electrode integration, the RE and CE electrodes were carefully inserted into the viscous polymer formulation shortly after insertion of the WE, and prior to complete polymer drying and dry‐state crosslinking. For the MN‐WE/RE/CE devices, the WE diameter was reduced from 6 mm to 4 mm to facilitate the positioning and separation of the 3 individual electrodes. A 3D printed holder, shown in Figure 7B, was used to guide the electrical wire connections and facilitate device application on the skin. Similarly to the MN‐WE arrays, digital microscopy analysis revealed excellent shape fidelity, except for the beneficial pencil‐sharpening effect, and a high level of reproducibility (Figure S22 and Table s16, Supporting Information).

The integrated MN‐WE/RE/CE (DS = 62%) devices presented the same general electrochemical and bioelectrocatalytic characteristics as the MN‐WE (DS = 62%) devices (Table S17, Supporting Information), accounting for the different geometric surface areas of the WEs. For example, the steady–state catalytic current density in 0.1 mol L^−1^ PB pH 7.4 for MN‐WE/RE/CE (ø = 4 mm) was 0.29 ± 0.03 mA cm^−2^ compared to 0.26 ± 0.02 mA cm^−2^ for MN‐WE (ø = 6 mm). Figure 7C highlights the attractive “fast” redox mediator response (*ΔE*p ≤ 36 ± 4) recorded in the artificial ISF‐containing soft skin model (4 wt% gelatin and 1 wt% agar) in the absence of glucose. Figure 7D reveals the well‐defined steady–state catalytic glucose oxidation current (0.24 ± 0.03 mA cm^−2^) obtained in the presence of 0.1 mol L^−1^ glucose in the artificial ISF‐containing soft skin. The electrochemical and bioelectrocatalytic parameters obtained in 0.1 mol L^−1^ PB and artificial soft skin are summarized in Tables S17 and S18 (Supporting Information), respectively.

The glucose biosensing performance of the MN‐WE/RE/CE device was evaluated for glucose concentrations ranging from 50 µmol L^−1^ up to 250 mmol L^−1^ in 0.1 mol L^−1^ PB (Figure S23 and Table S19, Supporting Information). Well‐defined hyperbolic plots were obtained. The catalytic current outputs are smaller compared to the equivalent nonintegrated BP_PLQ_‐GDH electrode. A comparison of the analytical sensor parameters, summarized in Table S19 (Supporting Information), reveals an increase in the biosensor stabilization time by a factor of 4 due to MN integration, a beneficial large improvement in the linear dynamic range from 25–30 to 25–50 mmol L^−1^, the same limit of detection of 0.1 mmol L^−1^, and an important decrease in the sensitivity down to 10.1 ± 0.9 µA mmol L^−1^ cm^−2^. These results underpin the important flux‐limiting behavior and bioelectrocatalytic compatibility of the highly crosslinked Dex‐MA (DS = 62%) MN matrix that offers reasonable performance in terms of sensitivity, limited performance in terms of stabilization time, and an attractive linear dynamic range.

Transdermal glucose monitoring with the MN‐WE/RE/CE (Dex‐MA DS = 62%) devices was demonstrated in both soft and hard artificial skin models containing artificial ISF (Figure 7E). In the presence of 5 mmol L^−1^ glucose, CGM was successfully achieved for 9.2 ± 0.6 days in soft skin and 10.6 ± 0.8 days in the artificial ISF‐containing hard skin model (24 wt% gelatin: 1 wt% agar). In the absence of glucose, the current outputs were negligible, confirming that the current originated from the bioelectrocatalytic oxidation of glucose extracted from the skin. The catalytic currents obtained in the ISF‐containing skin models were smaller compared to those obtained in bulk artificial ISF solution. This is primarily attributed to the mass transport‐limiting behavior of the skin that increased as the gelatinous content increased from 5% to 25%.

Two methods of calibration were developed based on either (i) the raw current output from periodic current–time plots, or (ii) the linear portion of the decay slope of the current–time CGM response. Examples of the CA data and corresponding calibration plots for glucose quantitation in the range of 1–25 mmol^−1^ are shown in Figure 7F–I. A summary of the extracted raw current and current slope values obtained at different glucose concentrations is available in Table S20 (Supporting Information). The “pre‐heating time” of 4.0 ± 0.3 h for the MN‐WE/RE/CE devices in artificial ISF‐containing soft skin was similar to the 4.5 ± 0.5 h exhibited by the single bioelectrode MN‐WE devices in artificial ISF solution. In control experiments, the specific glucose lag time in the soft skin model was estimated as 32 ± 5 min. This lag time, which corresponds to the time necessary for the equilibrium‐swollen hydrogel MN device to deliver 90% of the glucose oxidation response, is significantly larger that the < 5 min lag times reported for solid MNs, and similar to the lag‐time reported for passive‐diffusion hollow MN devices (20 min).^[^ ^37^ ^]^ The lag times are affected by factors such as the needle morphology and density, electrode structure and position with respect to the needles, hydrogel permeability, and the properties of the ISF (which will vary from person to person). Post‐mortem digital microscopy analysis was performed to inspect the needles of the MN‐WE/RE/CE devices after 10 days of CGM, and subsequent drying, as shown in Figure S24 (Supporting Information). No significant difference in the morphology of the MNs was observed compared to pristine MN devices, confirming the resistance of the Dex‐MA‐based MN arrays for long‐term continuous transdermal monitoring.

### 2.5 In Vitro Cytotoxicity Assessment of Electrodes and MN Devices

**Figure 8:**
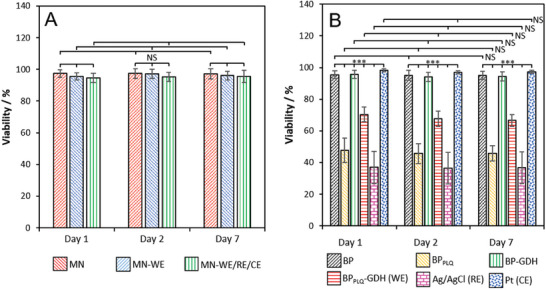
Cell viability of NIH3T3 murine fibroblasts in cell culture media after incubation for 1–7 days with A) microneedle devices: MN, MN‐WE and MN‐WE/RE/CE, and B) diverse electrodes and controls: BP unmodified, BP_PLQ_, BP‐GDH, BP_PLQ_‐GDH (WE), Ag/AgCl wire (RE), and Pt wire (CE). Results based on WST‐1 assay according to the ISO‐10993‐5 standard. The data is presented as mean ± SD (*n =* 15). Statistical significance was calculated using one‐way ANOVA; NS, ≥ 0.05, ^***^ *p* < 0.005.

One of the biggest barriers to the real‐world application of in vivo medical devices comes from the strict requirements in terms of biocompatibility and safety. Tests for in vitro cytotoxicity using murine NIH3T3 fibroblasts in cell culture media were performed according to the ISO‐accredited method for evaluating medical device biocompatibility (ISO 10993–5X; WST‐1 assay). The cytotoxicity of the fully integrated hydrogel MN biosensor devices, as well as their individual components, was evaluated. All crosslinked Dex‐MA materials were prepared using DS = 37% with 20 wt% polymer and 1% LAP formulations. **Figure** **8**A reveals cell viabilities in excess of ≥ 94.5% for MN devices prepared in the absence (MN) and presence of integrated electrodes (MN‐WE and MN‐WE/RE/CE) over 7 days. The high biocompatibility observed for the nonintegrated and electrode‐integrated MN hydrogel devices is attributed to the biocompatibility of the polymer as well as the barrier properties of the MN hydrogel that prevented or mitigated leaching of toxic components from the electrodes. A second series of experiments, shown in Figure 8B, unraveled the nature of the cytotoxicity of the three‐electrode bioelectrochemical sensor. Notably, the Dex‐MA hydrogel MN structure provides a remarkable cytotoxicity protection effect. The unmodified buckypaper (BP) and enzyme‐only modified buckypaper (BP‐GDH), and the Pt wire, exhibited high biocompatibility with cell viabilities of ≥ 95% over 7 days. On the other hand, the BP_PLQ_‐GDH (WE) electrode showed weak cytotoxicity with 70.3% viability (day 1) and ≥ 66.7% (day 7). The quinone‐only modified buckypaper (BP_PLQ_) exhibited higher levels of toxicity with 47.5% and 45.7% cell viabilities on day 1 and day 7, respectively. The toxicity under these conditions therefore originates from the quinone redox mediator as opposed to the redox enzyme or CNTs. The Ag/AgCl pseudo‐reference exhibited strong cytotoxicity, as expected, with ≈36.5% cell viability that can be attributed to delaminated AgCl and Ag^+^ ions with known toxicity to fibroblast cells.^[^ ^55^ ^]^ These first biocompatibility tests provide compelling evidence of “toxicity‐shielding” owing to the chemically crosslinked Dex‐MA hydrogel matrix that effectively prevents and/or limits electrode delamination and the leaching of toxic species.

## 3 Conclusion

The proof‐of‐concept is demonstrated for minimally invasive transdermal bioelectroenzymatic sensor devices that enable interstitial fluid extraction for selective and long‐term transdermal biomarker monitoring for 10 days in/on an artificial skin model containing artificial ISF. A low potential catalytically powerful second generation buckypaper 3‐electode biosensor was integrated into a dextran‐MA hydrogel microneedle matrix via a simple dual‐state enzyme‐compatible micromolding process. An eco‐friendly dextran methacrylation reaction was developed that enabled precise control over the degree of substitution to obtain a wide range of crosslinked hydrogels. The degree of substitution and consequent crosslinking density play a crucial role on the MN swelling behavior, the electroactivity, and the bioelectrocatalytic reactivity of the interpenetrated enzyme electrode. The ability to modulate the physical properties of the hydrogel materials permitted the long‐term monitoring stability to be further enhanced. The CGM performance pushes significantly beyond the state‐of‐the‐art transdermal bioelectroenzymatic sensors with demonstrated operational stability of ≤ 16 h. The long preheating times on the order of several hours, and ≈30 min glucose lag time, are linked primarily to mass transport and porous electrode wetting limitations. The preheating time is an important drawback. It would ideally be in the 0.5–2 h range, and could be improved with electrode integration closer to the needle tips. Ideally, the lag time would correspond to the “physiological lag time” only, on the order of 2–10 min. The lag time could be minimized, for example, by using a more permeable hydrogel to the ISF and glucose and/or by minimizing the physical distance and therefore mass transport distance between the needles and the electrode surface. The introduction of large pores, e.g., macropores or microchannels could be used to facilitate mass transport while surface functionalization combined with pore engineering could improve electrode wettability and stabilization.

As well as the flux‐limiting membrane properties, the hydrogel MNs exhibited a remarkable cytotoxicity shielding effect that allows the integration of working and reference electrodes presenting cytotoxicity. In the next step, to more convincingly validate the MN‐biosensor device in a real‐world setting, including extraction efficacy, the devices should be tested and validated in vivo versus existing glucometers. Indeed, live animals or humans motion may provoke sensor insertion, extraction, and stability complications. Ultimately, human trials will be necessary to fully validate the system. Further in vivo and in vitro cytotoxicity and biocompatibility studies, including a demonstration of infection risk, should be performed while the lag‐time limitations should be improved. The integration of alternative biosensor electrodes is envisaged toward the development of transdermal biosensors or “biowearables” for biomarkers beyond glucose. The microneedle system could offer promise as part of a self‐regulated system with transdermal insulin delivery.

## 4 Experimental Section

### Materials

Dextran T70 (Dex, M_w_ = 70 000 g mol^−1^) was purchased from Pharmacosmos. Methacrylic anhydride (MAh, 94%), lithium phenyl‐2,4,6 trimethyl benzoyl phosphinate (LAP, ≤ 95%), potassium chloride (KCl, >95%), N,N‐dimethylformamide (DMF, 99.9%), 1,10‐phenanthroline‐5,6‐dione (PLQ, 97%), gelatin from porcine skin (gel strength 300, Type A), agar, ethylenediaminetetraacetic acid (EDTA, ≥97.0%), bovine serum albumin (BSA, ≥ 95%), d‐(+)‐glucose (≥99.5%), sodium L‐lactate (98%), acetaminophen (98%), cholesterol (99%), urea (≤ 99%), D‐galactose (99%), L‐ascorbic acid (≤ 99%), and uric acid (≤ 99%) were purchased from Sigma–Aldrich. Sodium hydroxide (NaOH, ≥ 98%) was purchased from Laurylab. Distilled water was obtained by water purification to a resistivity of 15 MΩ cm using a Millipore Ultrapure system. Phosphate buffer (PB) (0.1 mol L^−1^) was prepared by dissolving NaH_2_PO_4_ in distilled water and adjusting the pH to 7.4 with NaOH. Phosphate buffer saline (0.01 mol L^−1^) (PBS containing 137 mmol L^−1^ NaCl, 2.7 mmol L^−1^ KCl, 10 mmol L^−1^ Na_2_HPO_4_, 1.8 mmol L^−1^ KH_2_PO_4_, pH 7.4) was obtained via a tenfold water dilution of PBS 10X (Sigma–Aldrich). Artificial interstitial fluid (ISF) was prepared by solubilizing 22 g L^−1^ of BSA and 0.3 g L^−1^ of EDTA in 0.01 mol L^−1^ PBS and adjusting the pH to 7.4 using NaOH.^[^ ^2^, ^56^ ^]^ Commercial grade multiwalled carbon nanotubes (CNTs, Ø = 9.5 nm, 1.5 µm length, ≥ 95% purity) were obtained from Nanocyl and used as received. FAD‐GDH (1160 U mg^−1^) was purchased from Sekisui. NIH‐3T3 murine fibroblast cells were purchased from the American Type Culture Collection (ATCC). WST‐1 was purchased from Roche Diagnostics. High purity argon was obtained from Messer. Glucose solutions were left to mutarotate overnight to β‐d‐glucose prior to use.

### Synthesis of Dextran‐Methacrylate (Dex‐MA)

Dex (5 g) was dissolved in distilled water (100 mL) in a beaker. After complete dissolution, different equivalents of MAh (0.0625–0.5 eq.) with respect to the number of Dex hydroxyl groups were added dropwise into the Dex solution. The solution was stirred at room temperature (RT) for 1 h. The pH was maintained at pH 9–11 using NaOH (3 mol L^−1^) during the reaction. Finally, the obtained Dex‐MA solution was dialyzed against distilled water for 1 week using a 12–14 kDa molecular weight cut‐off membrane (Carl Roth), and lyophilized. The white solid was stored at −20 °C then thawed to room temperature prior to use.

### Characterization of Dextran‐Methacrylate (Dex‐MA) Materials

FTIR spectroscopy was performed with a Shimadzu IR Affinity‐1 spectrophotometer with a DLATGS detector under a dry atmosphere at room temperature with lyophilized samples compressed against a KBr disk. ^1^H NMR spectra were recorded in D_2_O using a Bruker Avance 300 spectrometer at 298°K at 300 MHz. UV–vis absorption spectra were recorded between 200 and 800 nm using a Varian Cary 300 spectrophotometer at 25 °C in a quartz cell with a 1 cm path length. High resolution optical characterization was performed by digital microscopy using a VHX‐7000 microscope from Keyence.

### Preparation of Crosslinked Dex‐MA Hydrogels

PBS solutions (0.01 mol L^−1^) comprising a mixture of Dex‐MA at 10, 20, or 30 wt% polymer and LAP at 0.1, 0.5, or 1 wt% mass fraction were prepared. The solutions were cast into a Teflon 1 cm^3^ mold and left to dry in air for 24 h. Each mold contained the same dried polymer mass. After drying, the mixture was exposed to 405 nm UV‐light (UVAHand, Eleco‐Panacol, 75 mW cm^2^) for 60 s to obtain the dry crosslinked hydrogel. Swollen hydrogels were obtained by equilibrating the dried crosslinked pellets for 24 h in 0.01 mol L^−1^ PBS.

### Determination of Swelling Ratio and Insoluble Mass Fraction

These experiments were performed on crosslinked Dex‐MA hydrogels prepared using different polymer and LAP concentrations. Dried pellet samples were weighed (*W*_0_) after photocrosslinking. The samples of ≈20 mg mass were then incubated in a vial containing 1 mL of 0.01 mol L^−1^ PBS at RT.

### Swelling Ratio (SR)

Samples were periodically removed from the vial, quickly and carefully wiped with a paper towel to remove excess liquid, immediately weighed (*W*_t_), then placed back into the vial containing 0.01 mol L^−1^ PBS. This operation was repeated until no further mass change was detected (≤24 h). The percentage swelling ratio was calculated according to Equation (1):

(1) $$SR\left(\%\right)=\frac{W_{t}-\;W_{0}\;}{W_{0}}\times 100$$

### Insoluble Mass Fraction (IM)

Samples were incubated in 1 mL of 0.01 mol L^−1^ PBS at 37 °C for 1 week. After 1 week, samples were removed, dried completely in an oven at 80 °C, then weighed to obtain the insoluble weight (*W*_insoluble_). The percentage of IM was calculated according to Equation (2):

(2) $$IM\left(\%\right)=\frac{W_{\mathrm{insoluble}}}{W_{0}}\times 100$$

### Compressive Testing

Compression tests were performed using a TAXT.Plus texturometer (Stable Micro Systems) on dry hydrogels prepared using different polymer and LAP concentrations. The compression speed was set at 0.5 mm s^−1^ with a maximum compression force of 50 N. The compression modulus (*E*) was calculated from the linear section of the stress–strain curves between 5% and 15% strain. Three experiments were performed for each sample, and results are expressed as the mean value ± standard deviation.

### Cytotoxicity Evaluation

NIH‐3T3 fibroblasts were cultured under a humidified atmosphere (90%) comprising 95% air and 5% CO_2_ at 37 °C in culture medium with a high glucose content (Eagle medium with 10% fetal bovine serum and 1% of antibiotics (penicillin and streptomycin) (Dubelco).

Cytotoxicity tests were carried out according to the ISO‐10993‐5 standard on (i) buckypaper electrodes and (ii) the MN devices and their controls. Experiments were conducted using three different cell cultures in quintuplicate. All samples were sterilized in an ethanol (70%)/water (30%) mixture for 3 h. The ethanol/water solution was then removed and replaced twice by sterile 0.01 mol L^−1^ PBS with 1 h of soaking each time, for ethanol elimination. The samples were then incubated overnight for ≈16 h to achieve equilibrium swelling. The materials were set in a 24‐well plate and immersed in 1 mL of culture medium (3 cm^2^ of material for 1 mL of cell culture medium) for 24 h, 48 h, and 7 days. Control plates without materials, and cell culture medium only, were used as negative controls. The culture media (release media) were then collected in Eppendorf tubes after 24 h, 48 h, and 7 days, then frozen in a freezer at −20 °C. The cells were seeded with a cell density of 5000 cells per well in a 96‐well plate for 24 h at 37.5 °C with 5% CO_2_. After 24 h, the growth medium was replaced by 100 µL of material release medium after thawing at 37.5 °C, or by negative control medium, or cell culture medium with 10 mmol L^−1^ H_2_O_2_ as the positive cytotoxicity control. After 24 h, the cell culture media were removed and replaced with 100 µL of fresh cell culture medium and 10 µL of the WST‐1 reagent. After 2 h incubation at 37.5 °C with 5% CO_2_, the cell viability was calculated by reading the absorbance at 450 nm, with absorbance correction at 650 nm, with an Infinite‐M1000 Tecan microplate reader according to the following equation:

(3) $$\begin{array}{ccc} & & \mathrm{Cell}\;\mathrm{viability}\left(\%\right)\\ & & \;=\frac{\mathrm{Absorbanc}e_{\mathrm{sample}}-\mathrm{Absorbanc}e_{\mathrm{positive}\;\mathrm{cytotoxicity}\;\mathrm{control}}}{\mathrm{Absorbanc}e_{\mathrm{negative}\;\mathrm{cytotoxicity}\;\mathrm{control}}-\;\mathrm{Absorbanc}e_{\mathrm{positive}\;\mathrm{cytotoxicity}\;\mathrm{control}}}\\ & & \;\times 100 \end{array}$$

### Preparation of PDMS Microneedle Mold from Al Master

A microneedle (MN) master mold was manufactured from aluminum by a micro‐milling process. The Al master was composed of a cylindrical array of 7 mm with 5 × 5 (25) pencil‐shaped MNs with heights of 800, 1000, or 1200 µm, a base width of 400 µm, and an edge‐to‐edge distance between the needles of 400 µm. An inverse soft MN mold was prepared by casting a mixture of PDMS and its curing agent (10: 1 weight ratio) into the master mold, repeat application of vacuum to remove air bubbles, and curing at 100 °C for 2 h. The master and inverse molds were subsequently cooled under air for a few hours. The inverse PDMS mold was then gently detached from the master mold. Images of the master and PDMS molds are available in Figures S4 and S3 (Supporting Information), respectively.

### Preparation of Dex‐MA MN Arrays

MN patches were fabricated by micromolding. First, a solution containing 20 wt% of Dex‐MA and 1 wt% of LAP in 0.01 mol L^−1^ PBS pH 7.4 was prepared in a 10 mL vial. The solution was then cast into a PDMS mold. The PDMS mold was placed in a steel support connected to a vacuum pump set to 10 mbar, and the solution aspirated for 2 h to fill the tips of the mold. The solution was left to dry for 24 h to solidify the polymer. The MN arrays were then exposed to 405 nm light for 60 s (75 mW cm^2^) to obtain dried crosslinked MNs, left in the dark for a few hours, then demolded.

### Preparation of Enzymatic Buckypaper Bioelectrode (BP_PLQ_‐GDH)

First, 66 mg of MWCNTs were dispersed in 66 mL of DMF in a 100 mL vial then sonicated in a water bath for 90 min, according to the previously reported procedure.^[^ ^7^ ^]^ The suspension was filtered through a PTFE filter using a vacuum pump, washed with distilled water, then left for 2 h under the extraction hood. After filtration, the resulting buckypaper was left to dry at room temperature. Approximately 200 µm thick carbon nanotube buckypaper (BP_CNT_) was gently detached from the filter paper then cut into individual electrodes with Ø = 6 mm, unless stated otherwise. The buckypaper electrodes were modified by first drop‐coating 20 µL of a PLQ solution (5 mmol^−1^) prepared in acetone/distilled water (volume ratio of 1/1), then allowed to dry for 10 min. Thirty microliters of an FAD‐GDH solution in 0.1 mol L^−1^ PB (10 mg mL^−1^) was deposited on the surface of the modified electrode, then the electrode was left to dry for a few hours. The electrical contact was made via a Ag‐plated Cu wire attached to the back of the electrode with carbon paste. After drying for 2 h, the back of the electrode was insulated with silicone paste and left to dry in air prior to use.

### Fabrication of MN Array with an Integrated Enzymatic Buckypaper Bioelectrode as the Working Electrode (MN‐WE)

First, a BP_PLQ_‐GDH bioelectrode (Ø = 6 mm) was prepared. A solution containing 20 wt% Dex‐MA and 1 wt% LAP in 0.01 mol L^−1^ PBS was prepared in a 10 mL vial. Hundred microliters of the solution was then cast into a PDMS mold. The PDMS mold was placed in a steel support connected to a vacuum pump set to 10 mbar, aspirated for 2 h to fill the tips of the mold, then left to dry for 2–3 h to obtain a more viscous solution. The bioelectrode was integrated into the MN array via immersion inside the viscous polymer solution, then the hydrogel array was left to dry for 24 h. The solid polymer formulation was crosslinked by a first irradiation at the back of the array at 405 nm for 60 s (75 mW cm^2^), then a second irradiation at 405 nm for 60 s (75 mW cm^2^) after demolding and flipping of the MNs. Finally, the electrical connections were made at the back of the bioelectrode.

### Fabrication of MN Array with a Fully Integrated Three‐Electrode Bioelectroenzymatic Sensor (MN‐WE/RE/CE)

First, a smaller BP_PLQ_‐GDH bioelectrode (Ø = 4 mm) was prepared to provide more space for the insertion of multiple electrodes in the same device. The electrode fabrication procedure was as described above but with 9 µL of PLQ (5 mmol L^−1^) and 13.5 µL of FAD‐GDH (10 mg mL^−1^). The WE was integrated as described. A Ag/AgCl wire pseudo‐reference electrode (RE, Ø = 0.2 mm) and Pt counter electrode (CE, Ø = 0.2 mm) were subsequently integrated by immersion in the viscous polymer, as for the WE, ensuring that the electrodes did not touch. The dried (solid) polymer formulation was subsequently irradiated in two steps, then the electrical connection made to the WE.

### Preparation of Artificial Skin Models

Solutions of gelatin of 4–24 wt%, and agar of 1 wt%, were prepared in 40 mL of 0.01 mol L^−1^ PBS or artificial ISF in a 100 mL vial. “Soft skin” refers to artificial skin prepared with 4 wt% gelatin and 1 wt% agar while hard skin refers to artificial skin prepared with 24 wt% gelatin and 1 wt% agar. Unless stated otherwise, the skin was prepared using artificial ISF. Homogeneous solutions were obtained by heating at 80 °C for 30 min under stirring. The solutions were then cast into Petri dishes (ø = 5 cm) to yield artificial skin layers with a thickness of ≈2 cm that were left to cool overnight before use.

### Electrochemical Characterization

Static (quiescent) cyclic voltammetry measurements were performed using a Biologic VMP3 Multi Potentiostat with EC‐lab software. A Princeton Applied Research PARSTAT‐MC‐PMC1000 potentiostat controlled by Versastudio software, operating in a Faraday cage, was used for cyclic voltammetry experiments performed under stirring (at a fixed revolutions per minute, rpm), and fixed potential chronoamperometry measurements. For hydrodynamic experiments, a magnetic stirrer bar was placed in the electrolyte and the electrochemical cell placed on an IKA C‐mag magnetic stirrer (a rotating electrode was not used). All experiments were performed with a three‐electrode system comprising a Ag/AgCl reference electrode (commercial or chlorinated Ag wire pseudo‐reference), a Pt counter electrode, and the buckypaper‐based working electrode. The measurements were performed in 0.1 mol L^−1^ PB (pH 7.4), 0.01 mol L^−1^ PBS, or artificial ISF at room temperature. The Ag/AgCl pseudo‐reference was prepared by chlorination of a silver wire in 0.1 mol L^−1^ HCl solution at 1.8 V for 5 s.

The background capacitance, *C*, was calculated according to the following equation:

(4) $$C=\frac{Q}{V}$$

where, *Q* is the average charge during the charge/discharge process (in coulombs) and *V* is the potential window (in volts). The charge was calculated as the integral of the nonfaradaic current of the electrodes normalized by the scan rate. The surface concentration of electroactive redox mediator, denoted Γ (in mol cm^−2^) was calculated according to the following equation:

(5) $$\Gamma =\frac{Q}{nFA}$$

where *Q* is the average charge obtained from the anodic peaks of the redox mediator (in coulombs), *n* is the number of electrodes (2 electrons), and *F* is Faraday's constant (96 485 C mol^−1^), and *A* is the geometric surface area of the electrode (in cm^2^). The charge was calculated as the integral of the peaks associated with the oxidative faradaic process normalized by the scan rate (20 mV s^−1^). The limit of detection was calculated as the concentration associated with 3 × the baseline noise (signal‐to‐noise) ratio in the absence of glucose. The stabilization time was determined as the time necessary to achieve a stable current after the addition of glucose.

### Statistical Analysis

Statistics data is reported as the mean ± standard deviation together with the sample number, calculated using the corresponding functions in Microsoft Excel. One‐way ANOVA statistical significance tests were determined using three or more groups of data with Origin 2020 software. For the swelling ratio, insoluble weight data, and mechanical tests, each experiment was performed in triplicate on three individual pellet samples, or artificial skin samples, without preprocessing of data. Microneedle dimensions were measured on raw digital images obtained from three different arrays after adjusting image contrast. For cytotoxicity data, each experiment was performed on five samples in triplicate on different days (*n* = 15). For the electrochemical data, experiments were performed in triplicate on three individual electrodes without preprocessing of data, unless stated otherwise. The CVs and fixed potential CAs reported in figures are representative examples presented as the raw data, plotted and analyzed using Origin 2020 software. Electrochemical surface coverage was obtained via peak integration with linear background subtraction using the Peak Analyzer function in Origin 2020 software.

## Conflict of Interest

Bastien Darmau, Andrew J. Gross and Isabelle Texier have patented ‘Procédé de fonctionnalisation du dextran par des (méth)acrylates et utilisation du dextran ainsi fonctionnalisé pour préparer un hydrogel'. (FR 2114492) and submitted 2 related further publications concerning hydrogel microneedles (confidential).

## Supporting information



Supporting Information

## Data Availability

The data that support the findings of this study are available in the supplementary material of this article.
