# Supplementary material for: Self‐Extracting Dextran‐Based Hydrogel Microneedle Arrays with an Interpenetrating Bioelectroenzymatic Sensor for Transdermal Monitoring with Matrix Protection
Source: Adv Healthc Mater. 2024 Nov 24;14(2):2403209. doi: 10.1002/adhm.202403209 (PMC11729986; doi:10.1002/adhm.202403209)
Supplement: Supplementary file 1 — Supporting Information [file ADHM-14-0-s001.docx]

#

# Supporting Information

Self-Extracting Dextran-Based Hydrogel Microneedle Arrays with an Interpenetrating Bioelectroenzymatic Sensor for Transdermal Monitoring with Matrix Protection

Bastien Darmau, Marta Sacchi, Isabelle Texier*, Andrew J. Gross*

B. Darmau, A. J. Gross

Department of Molecular Chemistry, Univ. Grenoble Alpes-CNRS, 38041 Grenoble, France

E-mail: [andrew.gross@univ-grenoble-alpes.fr](mailto:andrew.gross@univ-grenoble-alpes.fr)

B. Darmau, M. Sacchi, I. Texier

CEA, LETI, Univ. Grenoble Alpes, F-38054 Grenoble, France

E-mail: [isabelle.texier-nogues@cea.fr](mailto:isabelle.texier-nogues@cea.fr)

**NMR characterisation of methacrylated dextran**

^1^H NMR spectra in D_2_O were recorded on purified as-received dextran and Dex-MA polymers. DS values, defined as the amount of methacryloyl groups per 100 dextran glucopyranose residues, were determined by the integration of the proton signals at 5.8 ppm and 6.25 ppm, corresponding to the vinyl groups of the methacrylate groups (I_9_), and the protons between 3.4 – 4.2 ppm, corresponding to the protons of the dextran carbons (I_2_ – I_6_). The peaks observed at 5.1 ppm and 5.4 ppm correspond to the anomeric proton (I_1_) with α-1,6 linkage (96 %) and α-1,3 linkage (4 %), respectively. The methyl protons of the methacrylate groups (I_10_) are observed at 1.9 ppm. The spectra are free of signals arising from the MAh precursor and other impurities, indicating well purified polymers.


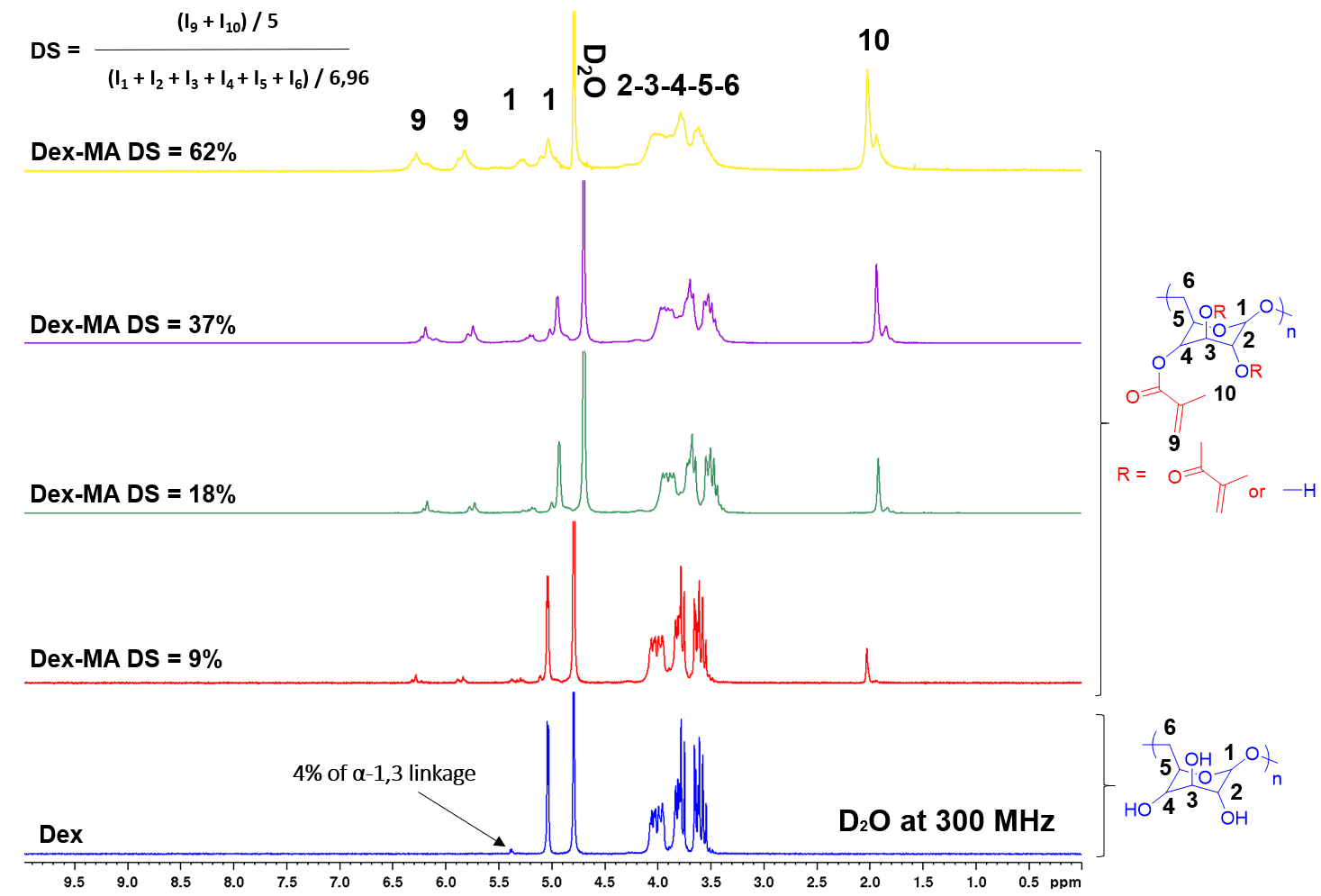


**Figure S1.** ^1^H NMR spectra in D_2_O at 300 MHz of purified dextran (Dex) and Dex-MA polymers with DS = 9 %, 18 %, 37 %, and 62 %. The corresponding structures and equation used to estimate DS are presented.


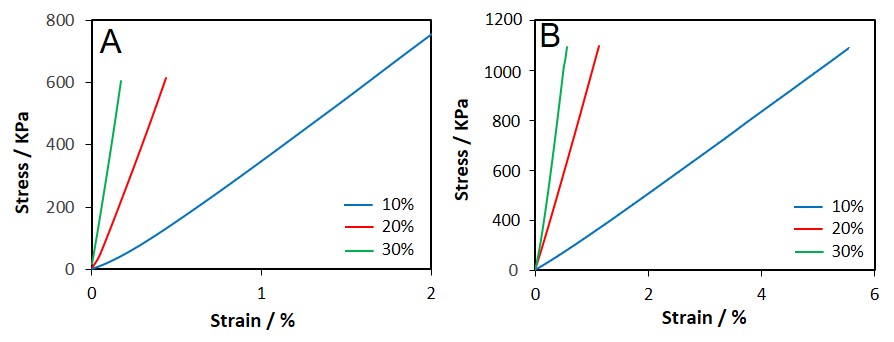


**Figure S2.** Stress-strain curves for dried crosslinked Dex-MA materials with (A) DS = 9 % and (B) DS = 37 % prepared with 10 – 30 wt% polymer in 0.01 M PBS pH 7.4 with 1 % LAP.

**Table S1.** Compression modulus (Young’s modulus) for Dex and crosslinked Dex-MA hydrogels in the dry state, prepared with 10 – 30 wt% polymer in 0.01 M PBS pH 7.4 with 1 % LAP. The data is presented as mean ± SD (*n =* 3).

| **Formulation** | **10 wt%** | **20 wt%** | **30 wt%** |
| --- | --- | --- | --- |
| Dex-MA (DS = 62 %) | 37 ± 2 MPa | 159 ± 7 MPa | 331 ± 10 MPa |
| Dex-MA (DS = 37 %) | 32 ± 2 MPa | 167 ± 10 MPa | 328 ± 13 MPa |
| Dex-MA (DS = 18 %) | 35 ± 2 MPa | 161 ± 9 MPa | 343 ± 11 MPa |
| Dex-MA (DS = 9 %) | 38 ± 2 MPa | 155 ± 7 MPa | 338 ± 8 MPa |
| Dex | 38 ± 2 MPa | 158 ± 3 MPa | 338 ± 5 MPa |


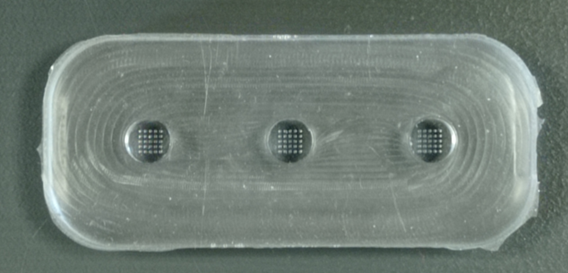


**Figure S3.** Photograph of a PDMS mold used to make 3 individual Dex-MA MN arrays.

**
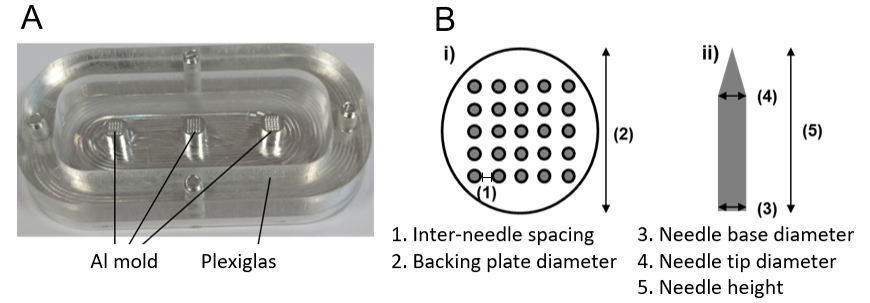
**

**Figure S4.** (A) Photograph of the machined Al master template fixed onto a plexiglass support. (B) (i) Top and (ii) side profile illustrations of the pencil-shape microneedle array design and the critical dimensions of the microneedle arrays.

**Table S2.** Summary of the characteristic dimensions of the Al master templates. The data is presented as mean ± SD (*n =* 3).

| **Design** | **MN height (µm)** | **MN base diameter**  **(µm)** | **MN top**  **diameter**  **(µm)** | **Inter-needle distance**  **(µm)** | **Base plate diameter**  **(µm)** |
| --- | --- | --- | --- | --- | --- |
| A | 790 ± 3 | 400 ± 1 | 398 ± 1 | 398 ± 1 | 7000 ± 1 |
| B | 978 ± 4 | 400 ± 1 | 398 ± 1 | 398 ± 1 | 7000 ± 1 |
| C | 1165 ± 6 | 400 ± 1 | 397 ±1 | 398 ± 1 | 7000 ± 1 |

**
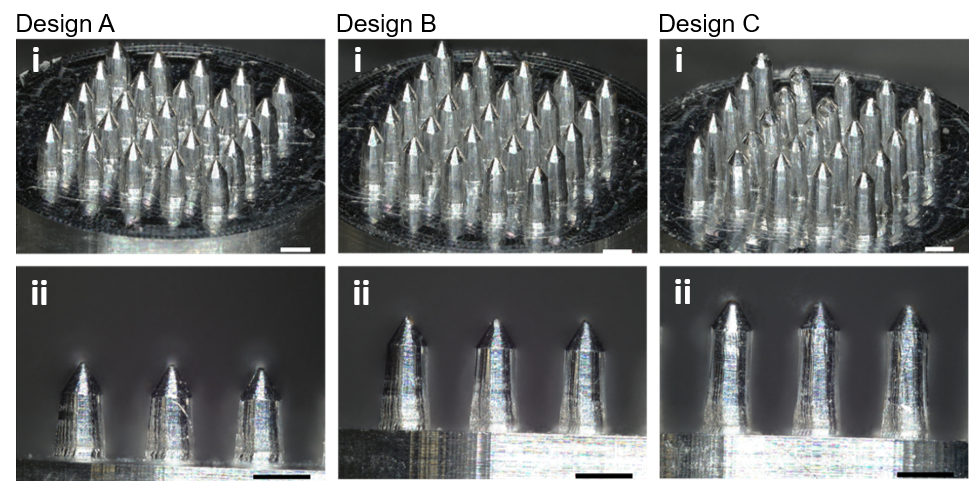
**

**Figure S5.** (A) High resolution digital images of the Al master templates: design A, B, and C with (i) top and (ii) side profiles. Scale bar = 500 µm.

**
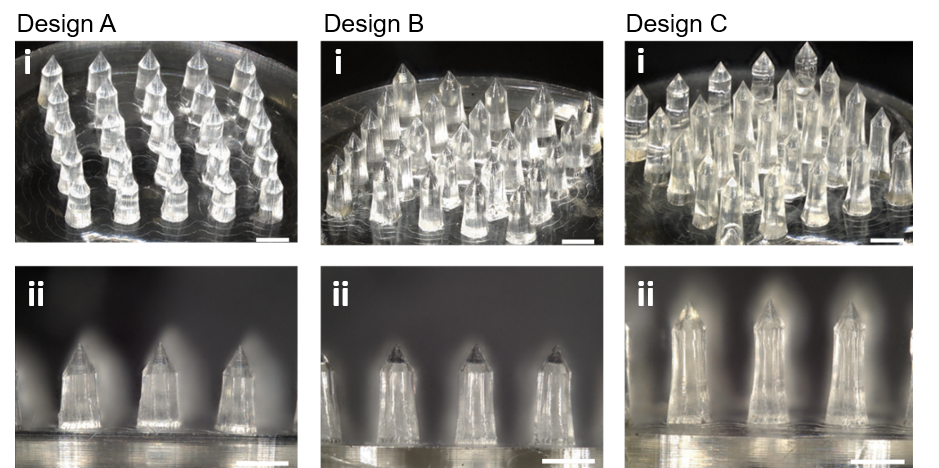
**

**Figure S6.** (A) High resolution digital images of Dex-MA hydrogel MN arrays (DS = 9%): design A, B, and C with (i) top and (ii) side profiles. Scale bar = 500 µm.

**Table S3.** Summary of the critical dimensions of the Dex-MA hydrogel MN arrays prepared with different DS and MN designs, based on high resolution digital images. The data is presented as mean ± SD (*n =* 3).

| **MN Design**  **(Dex-MA DS)** | **MN height (µm)** | **MN base diameter**  **(µm)** | **MN top diameter**  **(µm)** | **Inter-needle distance**  **(µm)** | **Base plate diameter**  **(µm)** |
| --- | --- | --- | --- | --- | --- |
| A (DS = 9 %) | 783 ± 4 | 395 ± 3 | 319 ± 2 | 385 ± 2 | 6724 ± 42 |
| B (DS = 9 % | 971 ± 7 | 396 ± 2 | 325 ± 3 | 386 ± 3 | 6709 ± 55 |
| C (DS = 9 %) | 1155 ± 10 | 396 ± 2 | 323 ± 3 | 384 ± 2 | 6725 ± 48 |
| A (DS = 18 %) | 782 ± 3 | 394 ± 2 | 329 ± 6 | 387 ± 2 | 6698 ± 78 |
| A (DS = 37 %) | 779 ± 3 | 389 ± 2 | 323 ± 4 | 383 ± 3 | 6732 ± 64 |
| A (DS = 62 %) | 782 ± 3 | 395 ± 2 | 322 ± 3 | 384 ± 2 | 6719 ± 49 |


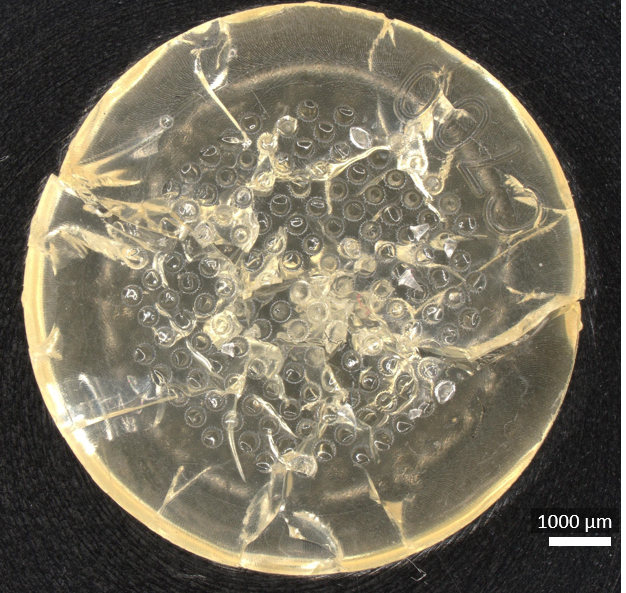


**Figure S7.** Typical example of an unsuccessful wet-state crosslinked Dex-MA (DS = 9 %, 20 wt%) MN array prepared and crosslinked in the presence of 1 % LAP in 0.01 M PBS pH 7.4.

**Table S4.** Summary of the characteristic dimensions and percentage expansion of Dex-MA hydrogel MN arrays prepared with different DS and MN designs, before (dry) and after swelling in 0.01 M PBS pH 7.4 for 2 h. Dimensions determined from high resolution digital images. The data is presented as mean ± SD (*n =* 3).

| **MN Design**  **(Dex-MA DS)** | **MN height (µm)**  **Dry \| Swollen**  **[ED%]** ^a)^ | **MN base diameter (µm)**  **Dry \| Swollen**  **[ED%]** | **MN top diameter (µm)**  **Dry \| Swollen**  **[ED%]** |
| --- | --- | --- | --- |
| A (DS = 9 %) | 790 ± 2 **\|** 915 ± 8  [15.8 ± 0.1] | 394 ± 2 **\|** 774 ± 12  [96.4 ± 1.6] | 335 ± 4 **\|** 462 ± 25  [37.9 ± 2.1] |
| B (DS = 9 % | 972 ± 2 **\|** 1120 ± 10  [15.2 ± 0.2] | 392 ± 3 **\|** 794 ± 8  [102.6 ± 1.1] | 328 ± 3 **\|** 462 ± 22  [40.9 ± 1.9] |
| C (DS = 9 %) | 1168 ± 4 **\|** 1345 ± 15 [15.2 ± 0.3] | 395 ± 3 **\|** 798 ± 7  [102.0 ± 1.2] | 320 ± 3 **\|** 434 ± 19  [35.6 ± 1.6] |
| A (DS = 62 %) | 788 ± 3 **\|** 796 ± 3  [0.6 ± 0.1] | 394 ± 2 **\|** 396 ± 6  [0.5 ± 0.1] | 321 ± 4 **\|** 324 ± 3  [0.9 ± 0.1] |
| B (DS = 62 %) | 967 ± 4 **\|** 977 ± 6  [1.0 ± 0.1] | 395 ± 2 **\|** 398 ± 8  [0.8 ± 0.1] | 322 ± 3 **\|** 325 ± 4  [0.9 ± 0.1] |
| C (DS = 62 %) | 1154 ± 3 **\|** 1162 ± 13  [0.7 ± 0.1] | 395 ± 2 **\|** 397 ± 6  [0.5 ± 0.1] | 318 ± 5 **\|** 322 ± 6  [1.0 ± 0.1] |

1. ED corresponds to the “expansion dimension” represented as the percentage change between the dry material and the wet material after equilibrium swelling in 0.01 M PBS pH 7.4.

**
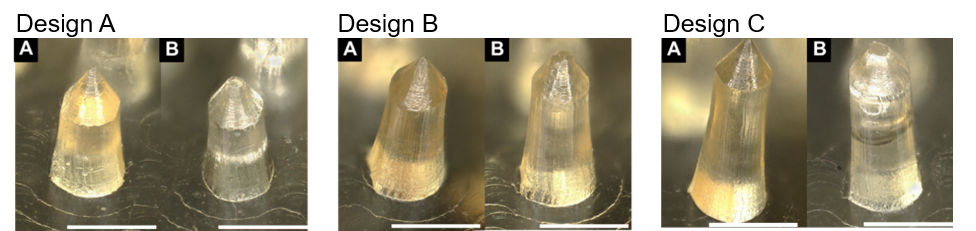
**

**Figure S8.** High resolution digital images of Dex-MA hydrogel MN arrays (DS = 37 %, 20 wt%): design A, B, and C obtained (A) before and (B) after compression with a vertical force of 50 N with a compression speed of 0.5 mm s^-1^. Scale bar = 500 µm.

**Table S5.** Summary of the characteristic dimensions and size reduction of dry Dex-MA hydrogel MN arrays (DS = 37 %, 20 wt%) before and after compression at 50 N with a compression speed of 0.5 mm s^-1^. Dimensions determined from high resolution digital images. The data is presented as mean ± SD (*n =* 3).

| **MN Design**  **(Dex-MA DS)** | **MN height (µm)**  **Dry \| Compressed \| [RD%]** ^a)^ | **MN top diameter (µm)**  **Dry \| Compressed \| [RD%]** ^a)^ |
| --- | --- | --- |
| A | 788 ± 3 **\|** 725 ± 10 **\|** [8.9 ± 1.6] | 500 ± 1 **\|** 498 ± 2 **\|** [< 1] |
| B | 972 ± 5 **\|** 916 ± 7 **\|** [5.9 ± 0.8] | 700 ± 1 **\|** 699 ± 1 **\|** [< 1] |
| C | 1152 ± 10 **\|** 1116 ± 12 **\|** [3.2 ± 1.1] | 900 ± 1 **\|** 899 ± 1 **\|** [< 1] |

^a)^ RD% corresponds to the “reduction dimension” represented as the percentage change between measurements recorded before and after compression with a vertical force of 50 N against a flat metal plate.

**Artificial skin model development and characterization**

The use of an artificial skin model overcomes the limitations associated with human and animal skin such as higher cost, limited availability, sample variability, and concerns regarding safety and handling.^[1]^ Nevertheless, there is no engineered artificial skin substitute that can replace all of the functions of intact human/animal skin and artificial models do present significant limitations. Care must be taken when comparing data obtained in artificial skin and human/animal skin. A common artificial skin surrogate for microneedle evaluation is based on agarose (0.4 – 1.8 %) since it is transparent, facilitating microscopic examination. Such models have an unrealistically high aqueous content of *ca.* 98 %. For example, Chang *et al.*, used an agarose skin model to mimic the dermis with ISF and demonstrate fluid swelling in hyaluronic acid crosslinked hydrogel MNs, monitored by optical coherence tomography, as a first step before validation on an animal model.^[2]^ Gelatin skin model formulations at 10 wt% have been proposed for transdermal microneedle evaluation, but the mechanical properties were not reported.^[3]^ We note that the fluid content of the human skin, including ISF present in the dermis, is generally considerered to be about 70 %.^[4,5]^ We used artificial skin models based on gelatin/agar mixtures that could (i) be cheaply and reproducibly obtained compared to real skin samples, (ii) be more realistic in terms of liquid content than agrose gels and therefore a more suitable model for electrochemical analysis, and (iii) previously demonstrated as effective skin mimics in terms of mechanical and other physical properties.^[6]^

We explored the preparation of artificial skin models based on 1 wt% agar. The gelatin concentration was tuned from 4wt% to 24wt% to match the mechanical properties of skin, such as the stiffness, and fluid content, up to a more realistic level of ca. 75% (Figure S9). The data clearly shows an increase in the compression modulus (stiffness) with increasing gelatin (solid) content. The obtained compression (Young’s) modulus values correspond well with the large range of values reported in the literature, e.g. 1 – 100 kPa^[6,7]^ and 0.01 – 2 MPa^[6,8,9]^ for artificial skin and human skin, respectively. In the report of Geerligs et al., values of 1 – 2 MPa were obtained via compression in the perpendicular direction at human epidermis, stratum corneum, and viable epidermis samples.^[9]^ On this basis, we employed two models: the hard skin model (24 wt% gelatin with 1 wt% agar) and the soft skin model (4 wt% gelatin with 1 wt% agar).

^
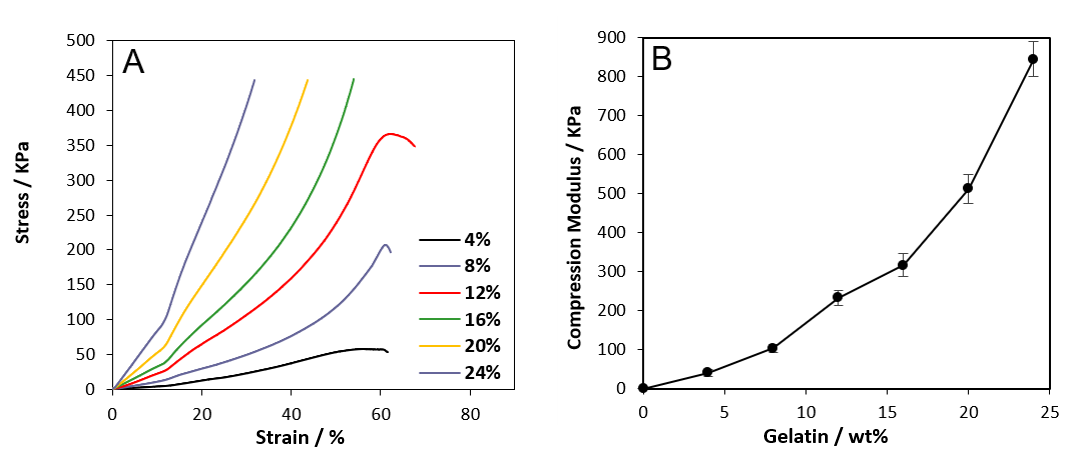
^

**Figure S9.** (A) Stress-strain curves obtained by compression at 0.5 mm s^-1^ on artificial skin models (5 cm diameter, 2 cm height) prepared with 1 wt% agar and varying amounts of gelatin (4 wt% to 24 wt%) in 0.01 M PBS, and (B) corresponding plot of the compression modulus as a function of the gelatin concentration. The compression modulus *vs.* gelatin concentration data is presented as mean ± SD (*n =* 3).

The skin models were prepared with artificial ISF comprising 22 g L^-1^ of albumin and 0.3 g of EDTA prepared in 0.01 M PBS pH 7.4 to better mimic human dermal ISF.^[10]^ The data in Table S6 summarises the characteristic mechanical properties obtained by compression tests (Figure S9), highlighting the similarity between artificial skin models prepared in 0.01 M PBS and artificial ISF in terms of the mechanical properties.

**Table S6**. Characteristic mechanical properties of artifical skin models containing either 4 wt% or 24 wt% gelatin with 1 wt% agar prepared in a solution of 0.01 M PBS or artifical ISF at pH 7.4. The data is presented as mean ± SD (*n =* 3)

| Skin model | Compression modulus (E) / KPa | Ultimate compression strength  (UCS) / KPa | Ultimate strain  (US) / % |
| --- | --- | --- | --- |
| 4 % PBS | 40 ± 9 | 62 ± 6 | 62.5 ± 3.9 |
| 4 % ISF | 54 ± 10 | 78 ± 4 | 59.4 ± 4.1 |
| 24 % PBS | 844 ± 45 | < 440 | < 31.8 |
| 24 % ISF | 947 ± 19 | < 440 | < 30.1 |


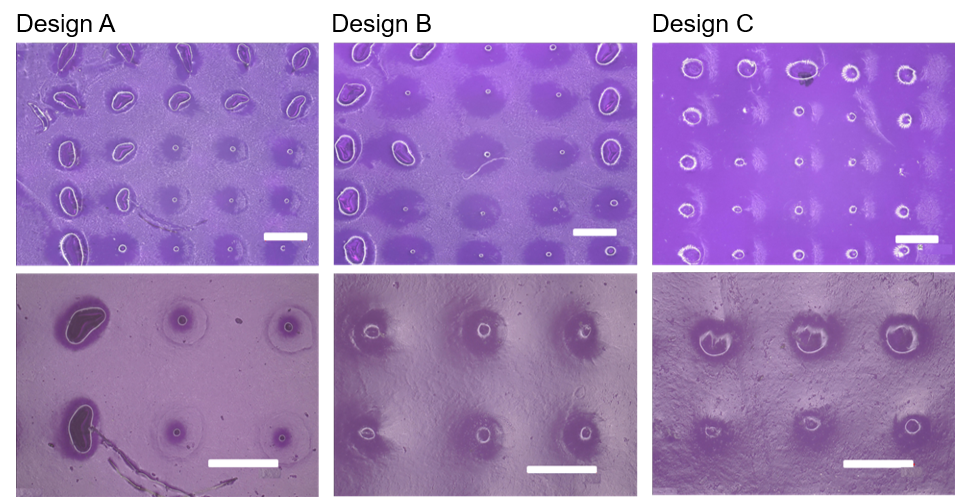


**Figure S10.** High resolution digital images of perforated “hard” artificial skin (24 wt% gelatin, 1 wt% agar) by Dex-MA hydrogel MN arrays (DS = 9 %, 20 wt%): design A, B, and C. Scale bar = 500 µm.


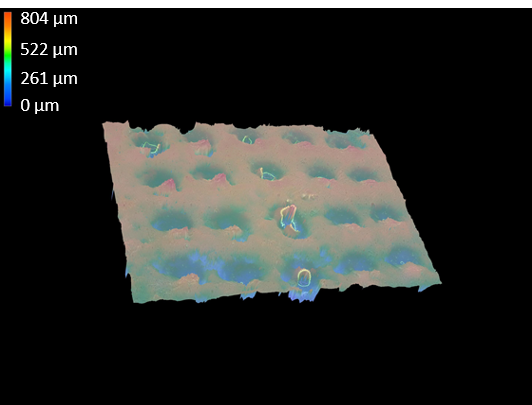


**Figure S11.** High resolution digital laser images of perforated “hard” artificial skin (24 wt% gelatin, 1 wt% agar) by Dex-MA hydrogel MN arrays (DS = 9 %, 20 wt%): design A.


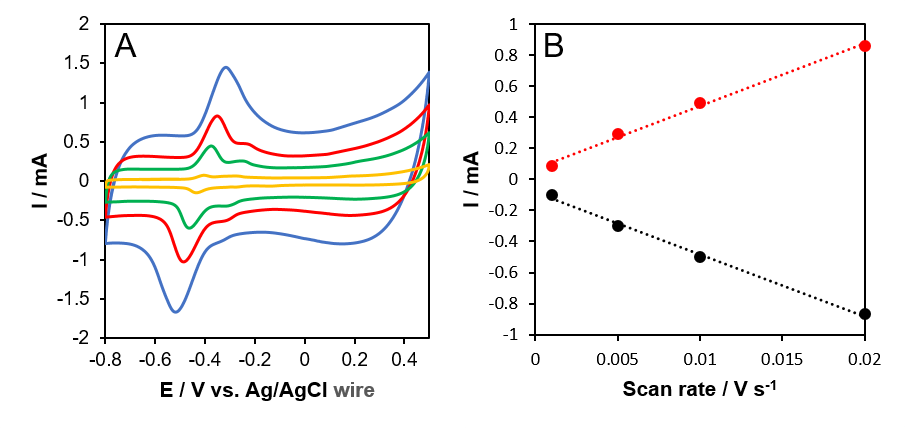


**Figure S12.** (A) CVs at BP_PLQ_ in 0.1 M PB pH 7.4 at varying scan rate (1 mV s^-1^ (yellow), 5 mV s^-1^ (green), 10 mV s^-1^ (red), 20 mV s^-1^ (blue) and the corresponding linear Randles-Ševčík plot (r^2^ > 0.99) of the peak current *vs.* scan rate.

**Table S7**. Comparison of the electrochemical parameters for BP_PLQ_ and BP_PLQ_-GDH obtained from CVs recorded in simple and complex buffers. The data is presented as mean ± SD (*n =* 3).

| Electrode | Buffer | *E_1/2_* / V  *vs.* Ag/AgCl wire | *ΔE_p_* / mV  (1 mV s^-1^) | Γ / mol cm^-2^  (20 mV s^-1^) | *Q* / mF  (20 mV s^-1^) |
| --- | --- | --- | --- | --- | --- |
| BP_PLQ_ | **0.1 M PB** | - 0.41 ± 0.03 | 25 ± 3 | 1.32 ± 0.07 × 10^-7^ | 75.1 ± 6.0 |
|  |  | -0.27 ± 0.02 | 28 ± 3 |  |  |
| BP_PLQ_-GDH | **0.1 M PB** | - 0.38 ± 0.02  -0.27 ± 0.03 | 72 ± 3  85 ± 5 | 1.32 ± 0.06 × 10^-7^ | 22.1 ± 1.7 |
| BP_PLQ_-GDH | **0.01 M PBS** | - 0.31 ± 0.02 | 78 ± 8 | 6.45 ± 0.06 × 10^-8^ | 20.9 ± 2.4 |
|  |  | -0.18 ± 0.02 | 145 ± 18 |  |  |
| BP_PLQ_-GDH | **ISF** | - 0.31 ± 0.01 | 80 ± 10 | 6.28 ± 0.07 × 10^-8^ | 21.1 ± 2.1 |
|  |  | -0.18 ± 0.02 | 154 ± 18 |  |  |

**Ag/AgCl pseudo-reference electrode characterization**

The potential of the Ag/AgCl wire pseudo-reference was evaluated in different media at pH 7.4: 0.1 M PB, 0.01 M PB with 137 mmol L^-1^ NaCl and 2.7 mmol L^-1^ KCl (commercial 1X PBS), and 0.1 M PB with saturated KCl in the presence of 0.25 mmol L^-1^ potassium ferricyanide at polished glassy carbon (GC) electrodes (⌀ = 3 mm). The values were compared with a commercial Ag/AgCl electrode (sat. KCl). Table S8 presents the *E*_1/2_ obtained for 0.2 mmol L^-1^ potassium ferricyanide in solution as a function of the reference electrode and buffer type.


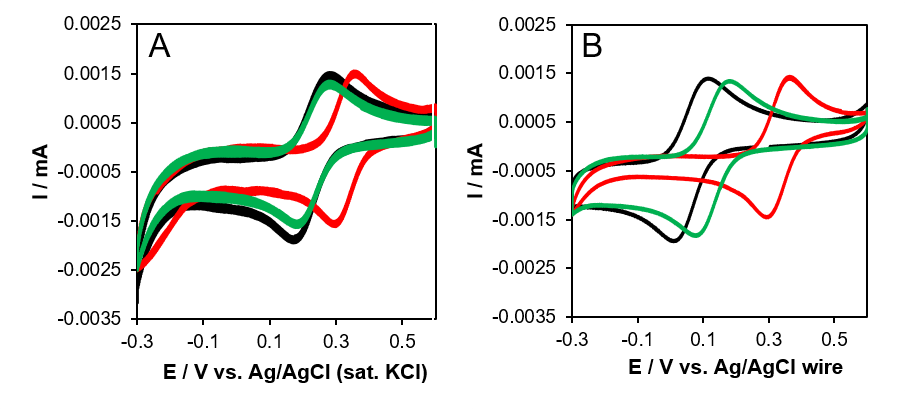


**Figure S13.** CVs recorded in 0.25 mmol L^-1^ potassium ferricyanide at GC at 20 mV s^-1^ in 0.1 M PB (black), commercial 0.01 M 1X PBS (green), and 0.1 M PB with saturated KCl (red). The CVs was obtained versus (A) a commercial Ag/AgCl reference (sat. KCl) and B) homemade chlorinated Ag/AgCl wire pseudo-reference electrode.

The *E*_1/2_ of the redox probe shifted by -0.17 V for the pseudo-reference electrode compared to the commercial Ag/AgCl (sat. KCl) reference electrode in simple 0.1 M PB. In contrast, practically no difference in potential was observed between the reference electrodes for the redox probe response obtained in saturated KCl solution. This reflects the high stability of the equilbrium Ag/AgCl reaction at both the pseudo and commercial reference electrodes in the presence of excess chloride. In 0.01 M 1X PBS, the *E*_1/2_ of the redox probe shifted by -0.08 V for the pseudo-reference electrode compared to the commercial Ag/AgCl (sat. KCl). The *E*_1/2_ obtained at commercial Ag/AgCl was essentially the same in 0.1 M PB and 0.01 M 1X PBS, hence the observed -0.08 V shift occured due to the Ag/AgCl wire pseudo-reference electrode whose potential varies according to the concentration of chloride ions in solution.^[11]^

**Table S8**. Half-wave and potential shift values determined from CVs recorded at GC at 20 mV s^-1^ in different buffer solutions containing 0.25 mmol L^-1^ potassium ferricyanide redox probe versus a commercial Ag/AgCl (sat. KCl) reference and a chlorinated Ag/AgCl wire pseudo-reference electrode.

| Buffer | *E_1/2_* / V  Commercial  Ag/AgCl (sat. KCl) | *E_1/2_* / V  Pseudo-reference  Ag/AgCl wire | Potential  shift */ V* |
| --- | --- | --- | --- |
| 0.1 M PB | 0.24 | 0.07 | -0.17 |
| 0.1 M PB with sat. KCl | 0.34 | 0.34 | 0 |
| 0.01 M 1X PBS | 0.23 | 0.15 | -0.08 |

**able S9.** Comparison of the bioelectrocatalytic glucose oxidation steady-state current for BP_PLQ_-GDH in simple and complex buffers before and after purging with argon in 0.1 M PB and artificial ISF at pH 7.4. The data is presented as mean ± SD (*n =* 3).

| Electrode | Buffer | Catalytic current density at 1 mV s^-1^  (mA cm^-2^) | |  |
| --- | --- | --- | --- | --- |
|  |  | Quiescent | Argon-saturated | |
| BP_PLQ_-GDH | 0.1 M PB | 1.68 ± 0.06 | 1.65 ± 0.06 | |
| BP_PLQ_-GDH | Artificial ISF | 0.46 ± 0.04 | 0.44 ± 0.03 | |


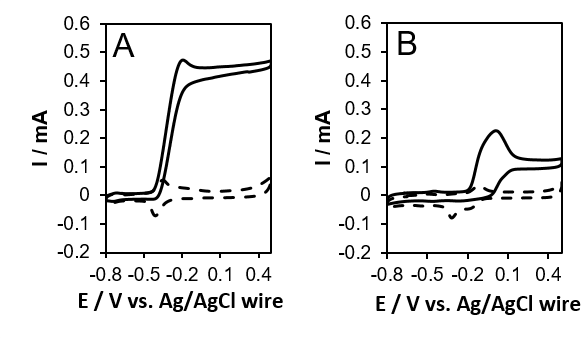


**Figure S14.** CVs at BP_PLQ_-GDH in argon-saturated (A) 0.1 M PB and (B) artificial ISF at pH 7.4 at 1 mV s^-1^ in the absence (dash) and presence (solid) of 0.1 mol L^-1^ glucose.

^
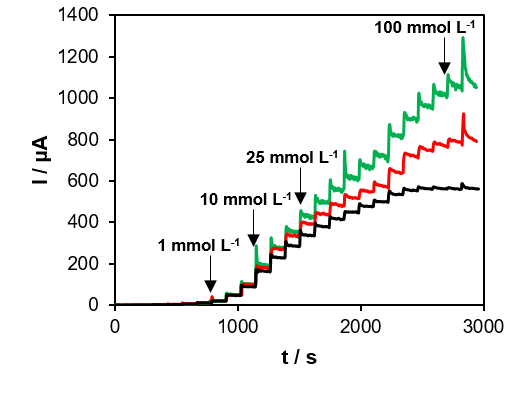
^

**Figure S15.** Fixed potential chronoamperograms recorded at BP_PLQ_-GDH in 0.1 M PB at increasing glucose concentrations at an applied potential of 0.1 V (green), -0.1 V (red), and -0.3 V (black) *vs.* Ag/AgCl pseudo-reference at 500 rpm.

**Table S10** Comparison of the bioelectrocatalytic glucose oxidation sensor performance parameters obtained from chronoamperograms performed in 0.1 M PB pH 7.4 at increasing glucose concentrations at different applied potentials under static (quiescent) or hydrodynamic (500 rpm) conditions. The data is presented as mean ± SD (*n =* 3).

| Type | Potential  (V *vs.* Ag/AgCl wire) | Linear range  (mmol L^-1^) ; r^2^ | Sensitivity  (µA mM^-1^cm^-2^) | Detection limit  (mmol L^-1^) | Response time (s) |
| --- | --- | --- | --- | --- | --- |
| 500 rpm | - 0.3 | 0.05 - 25 ; 0.993 | 68.6 ± 4,9 | 0.05 | 26.7 ± 8.5 |
| 500 rpm | - 0.1 | 0.05 - 30 ; 0.992 | 71.9 ± 4,5 | 0.05 | 24.9 ± 6.7 |
| 500 rpm | + 0.1 | 0.05 - 35 ; 0.995 | 72.3 ± 5,9 | 0.05 | 26.2 ± 8.1 |
| Static | - 0.3 | 1 – 50 ; 0,996 | 22.8 ± 1.2 | 0.2 | 47.7 ± 4.8 |
| Static | - 0.1 | 1 – 50 ; 0,997 | 25.3 ± 1.0 | 0.2 | 45.8 ± 4.8 |
| Static | + 0.1 | 1 – 50 ; 0,996 | 24.4 ± 1.1 | 0.2 | 45.1 ± 4.7 |


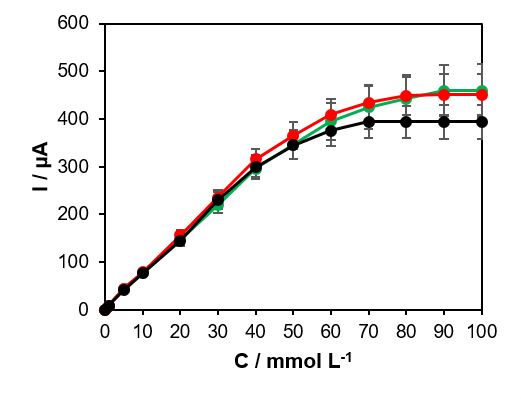


**Figure S16.** Glucose bioelectrocatalytic oxidation current *vs.* concentration plotted from fixed potential CA measurements recorded at BP_PLQ_-GDH in 0.1 M PB at 0.1 V (green), -0.1 V (red), and -0.3 V (black) *vs.* Ag/AgCl without agitation. The data is presented as mean ± SD (*n =* 3).

**Table S11.** Summary of performance parameters obtained from fixed potential CA experiments recorded at BP_PLQ_-GDH in artificial ISF in the absence and presence of additional potential interferents at different potentials. The composition includes: 0.01 M PBS 1X with 22 g L^-1^ BSA, 0.3 g L^-1^ EDTA, 0.2 mmol L^-1^ acetaminophen, 1 mmol L^-1^ cholesterol, 8 mmol L^-1^ urea, 2 mmol L^-1^ lactate, 0.3 mmol L^-1^ galactose, 0.5 mmol L^-1^ uric acid, and 0.1 mol L^-1^ ascorbic acid.^[12–16]^

| Applied Potential  (V *vs.* Ag/AgCl) ^a)^ | Adjusted Potential  (V *vs.* Ag/AgCl) ^b)^ | Interference Total  (%) | Current  1^st^ glucose addition  (µA) | Current 2^nd^ glucose addition  (µA) | Current decrease  (%) |
| --- | --- | --- | --- | --- | --- |
| - 0.3 | ≈ -0.22 | 25.1 | 92.3 | 22.6 | 75.5 |
| - 0.2 | ≈ -0.12 | 6.0 | 97.9 | 54.7 | 44.1 |
| -0.1 | ≈ -0.02 | 6.8 | 97.7 | 72.6 | 25.7 |
| 0 V | ≈ +0.08 | 11.1 | 96.4 | 88.2 | 8.5 |

^a)^ The actual potential applied for the experiments performed in artificial ISF with the pseudo-reference electrode.

^b)^ The estimated potential for an equivalent experiment performed in 0.1 M PB in the absence of chloride ions, accounting for the chloride-dependent potential shift observed for the Ag/AgCl wire pseudo-reference electrode.

^
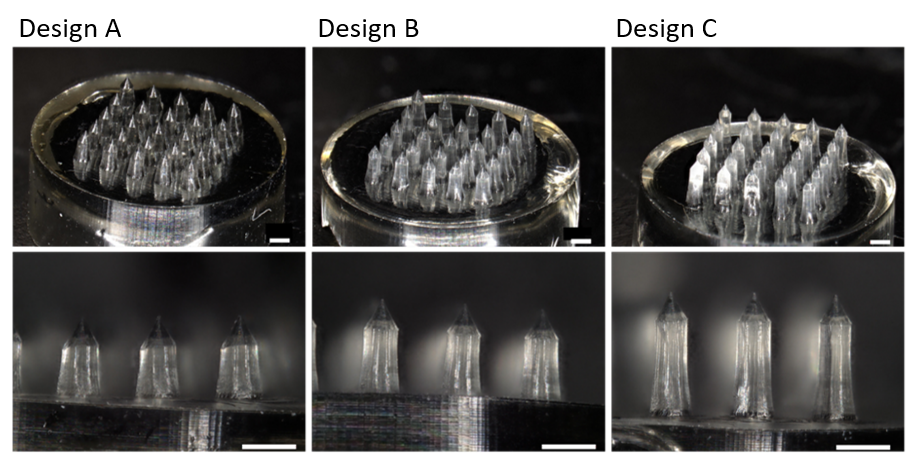
^

**Figure S17.** High resolution digital images of Dex-MA hydrogel MN arrays (DS = 62%, 20 wt%) with integrated BP_PLQ_-GDH (MN-WE): design A, B, and C. Scale bar = 500 µm.

**Table S12.** Summary of the characteristic dimensions of Dex-MA hydrogel MN arrays (DS = 62 %, 20 wt%) with integrated BP_PLQ_-GDH (MN-WE) prepared with different MN designs. The data is presented as mean ± SD (*n =* 3).

| **Design** | **MN height (µm)** | **MN base diameter**  **(µm)** | **MN top diameter (µm)** | **Inter-needle distance**  **(µm)** | **Base plate diameter (µm)** |
| --- | --- | --- | --- | --- | --- |
| **A** | 781 ± 3 | 394 ± 3 | 323 ± 3 | 384 ± 2 | 6712 ± 27 |
| **B** | 968 ± 3 | 392 ± 2 | 321 ± 3 | 387 ± 2 | 6719 ± 28 |
| **C** | 1149 ± 5 | 396 ± 2 | 323 ± 3 | 387 ± 2 | 6722 ± 35 |


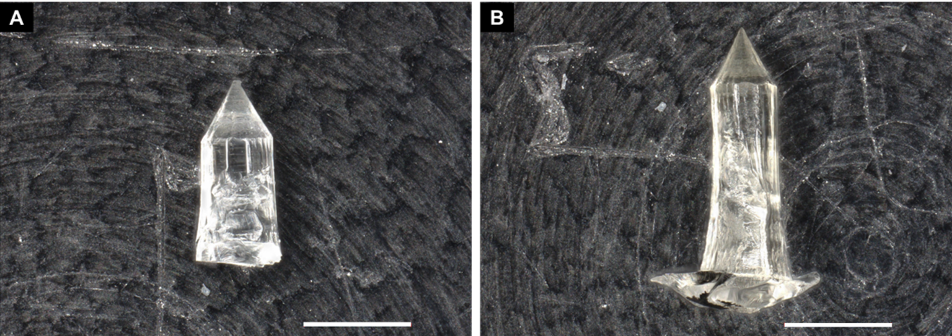


**Figure S18.** High resolution digital microscopy of individual Dex-MA hydrogel MNs removed from MN-WE device with a needle height of (A) 780 µm and (B) 1162 µm. Scale = 500 µm.

~~
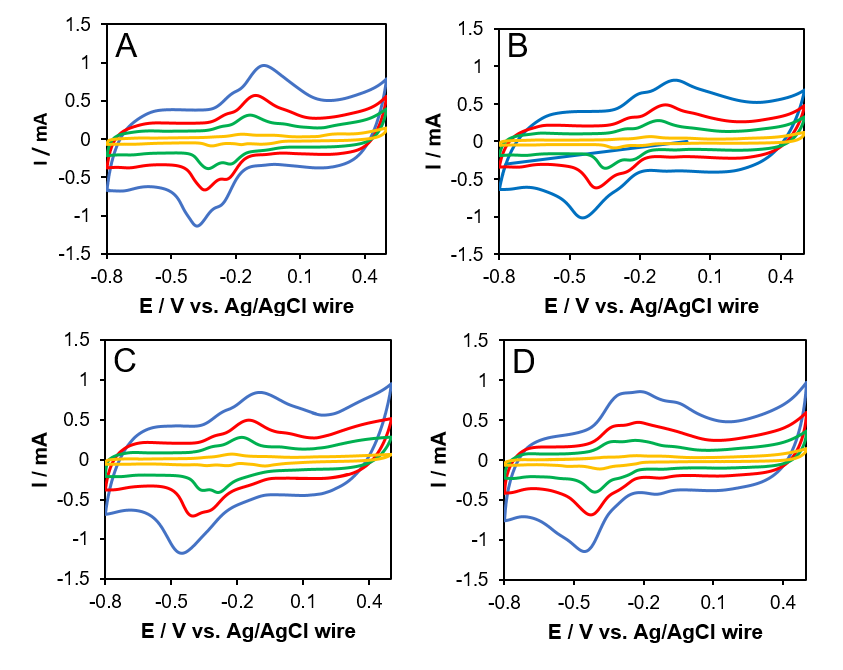
~~

**Figure S19.** CVs recorded in 0.1 M PB pH 7.4 at varying scan rate (1 mV s^-1^ (yellow), 5 mV s^-1^ (green), 10 mV s^-1^ (red), 20 mV s^-1^ (blue)) for crosslinked Dex-MA hydrogel MN-WE arrays with an integrated BP_PLQ_-GDH for Dex-MA: (A) DS = 9 %, (B) DS = 18 %, (C) DS = 37 %, and (D) DS = 62 % prepared with 20 wt% polymer.

**Table S13.** Estimated electrochemical parameters obtained from CVs recorded at 1 mV s^-1^ to 20 mV s^-1^ in bulk 0.1 M PB at pH 7.4 for crosslinked Dex-MA MN-WE (DS = 9% to 62%; 20 wt% polymer) with BP_PLQ_-GDH *vs.* non-integrated BP_PLQ_-GDH bioelectrodes. The data is presented as mean ± SD (*n =* 3).

| Electrode | Buffer | *E_1/2_* / V *vs.* Ag/AgCl wire | *ΔE_p_* / mV  (1 mV s^-1^) | Γ / mol cm^-2^  (20 mV s^-1^) | *Q* / mF  (20 mV s^-1^) |
| --- | --- | --- | --- | --- | --- |
| WE | PB | - 0.38 ± 0.02 | 72 ± 3 | 1.32 ± 0.09 × 10^-7^ | 22.1 ± 1.7 |
|  |  | - 0.27 ± 0.03 | 85 ± 5 |  |  |
| MN-WE  (DS = 9 %) | PB ^a)^ | - 0.30 ± 0.02  - 0.22 ± 0.03 | 40 ± 5  29 ± 5 | 1.51 ± 0.11 × 10^-7^ | 51.1 ± 3.8 |
| MN-WE  (DS = 18 %) | PB ^a)^ | - 0.29 ± 0.02  - 0.22 ± 0.03 | 41 ± 5  32 ± 4 | 1.39 ± 0.13 × 10^-7^ | 53.5 ± 4.5 |
| MN-WE  (DS = 37 %) | PB ^a)^ | - 0.31 ± 0.02  -0.23 ± 0.02 | 38 ± 4  33 ± 4 | 1.45 ± 0.10 × 10^-7^ | 52.9 ± 4.0 |
| MN-WE  (DS = 62 %) | PB ^a)^ | -0.29 ± 0.02  -0.23 ± 0.03 | 42 ± 4  33 ± 4 | 1.54 ± 0.15 × 10^-7^ | 54.2 ± 3.6 |

^a)^ The electrochemical experiments were performed in bulk PB solution but the local electrolyte in the MNs contains NaCl that originates from the initial polymer formulation prepared in 0.01 M PBS.


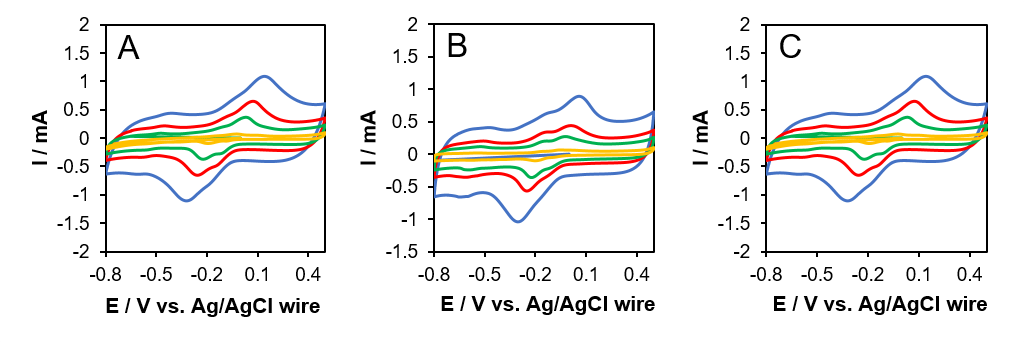


**Figure S20.** CVs at varying scan rate (1 mV s^-1^ (yellow), 5 mV s^-1^ (green), 10 mV s^-1^ (red), 20 mV s^-1^ (blue)) for Dex-MA hydrogel MN-WE arrays (DS = 37 %): design A with integrated BP_PLQ_-GDH in (A) 0.01 M PBS, (B) artificial ISF, (C) soft skin (4 wt% gelatin, 1 wt% agar) at pH 7.4.

**Table S14.** Estimated electrochemical parameters obtained from CVs recorded in 0.01 M PBS, artificial ISF, and artificial soft skin (4% gelatin, 1% agar) for crosslinked Dex-MA MN-WE (DS = 37%; 20 wt% polymer) with BP_PLQ_-GDH *vs.* non-integrated BP_PLQ_-GDH bioelectrodes. The data is presented as mean ± SD (*n =* 3).

| Electrode | Buffer | *E_1/2_* / V *vs.* Ag/AgCl wire | *ΔE_p_* / mV  (1 mV s^-1^) | Γ / mol cm^-2^  (20 mV s^-1^) | *Q* / mF  (20 mV s^-1^) |
| --- | --- | --- | --- | --- | --- |
| WE | PBS | -0.31 ± 0.02  -0.18 ± 0.02 | 78 ± 8  145 ± 18 | 6.45 ± 0.06 × 10^-8^ | 20.9 ± 2.4 |
| WE | ISF | - 0.31 ± 0.01  -0.17 ± 0.02 | 80 ± 10  154 ± 18 | 6.28 ± 0.07 × 10^-8^ | 21.1 ± 2.1 |
| MN-WE  (DS = 37 %) | PBS | - 0.18 ± 0.02  - 0.09 ± 0.02 | 39 ± 3  37 ± 2 | 1.37 ± 0.10 × 10^-7^ | 52.9 ± 4,0 |
| MN-WE  (DS = 37 %) | ISF | - 0.17 ± 0.02  -0.08 ± 0.02 | 43 ± 3  37 ± 3 | 1.42 ± 0.10 × 10^-7^ | 50.1 ± 5.3 |
| MN-WE  (DS = 37 %) | Soft skin | - 0.17 ± 0.01  - 0.07 ± 0.02 | 44 ± 3  35 ± 4 | 1.51 ± 0.1 × 10^-7^ | 53.8 ± 4.9 |

**Table S15**. Steady-state bioelectrocatalytic currents obtained at 0.3 V *vs.* Ag/AgCl wire from CVs recorded at 1 mV s^-1^ in 0.1 M PB, 0.01 M PBS, artificial ISF, and artificial soft skin (4% gelatin, 1% agar) for crosslinked Dex-MA MN-WE (DS = 9% to 62%; 20 wt% polymer) with BP_PLQ_-GDH *vs.* non-integrated BP_PLQ_-GDH bioelectrodes. The data is presented as mean ± SD (*n =* 3).

| Electrode | Current density at 1 mV s^-1^ / mA cm^-2^ | | | |
| --- | --- | --- | --- | --- |
|  | **PB** | **PBS** | **ISF** | **Soft skin**  **(4 wt% gelatin, 1 wt% agar)** |
| WE | 1.68 ± 0.06 | 0.47 ± 0.03 | 0.46 ± 0.04 | / |
| MN-WE  (DS = 9 %) | 0.58 ± 0.03 | / | / | / |
| MN-WE  (DS = 18 %) | 0.44 ± 0.03 | / | / | / |
| MN-WE  (DS = 37 %) | 0.31 ± 0.02 | 0.24 ± 0.02 | 0.23 ± 0.03 | 0.23 ± 0.03 |
| MN-WE  (DS = 62 %) | 0.26 ± 0.02 | / | / | / |


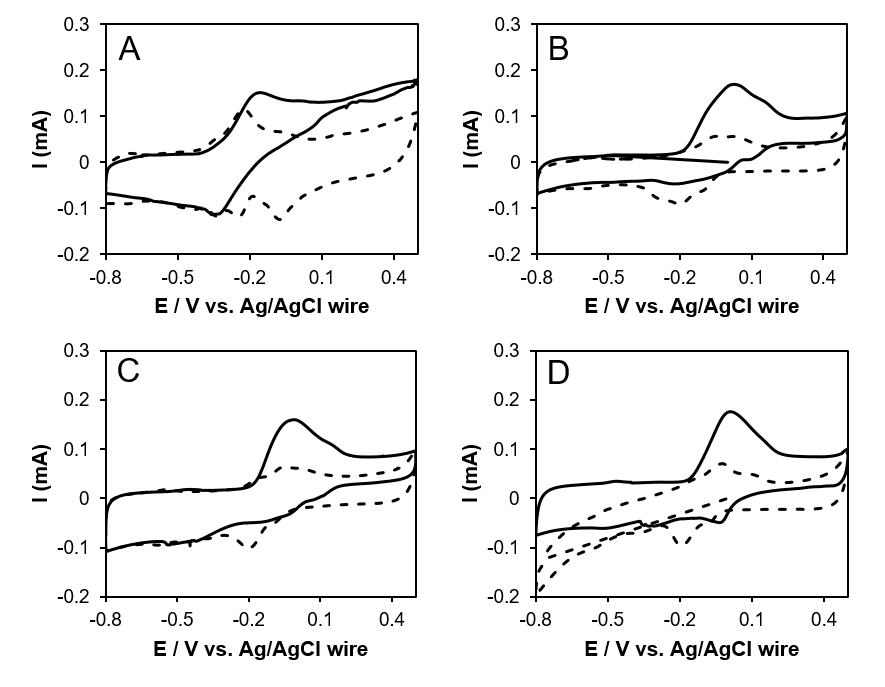


**Figure S21**. CVs in 0.1 M PB pH 7.4 at 1 mV s^-1^ in the absence (dash) and presence (solid) of 0.1 mmol L^-1^ glucose for Dex-MA hydrogel MN-WE arrays: design A with integrated BP_PLQ_-GDH for Dex-MA (A) DS = 9 %, (B) DS = 18 %, (C) DS = 37 %, and (D) DS = 62 %.


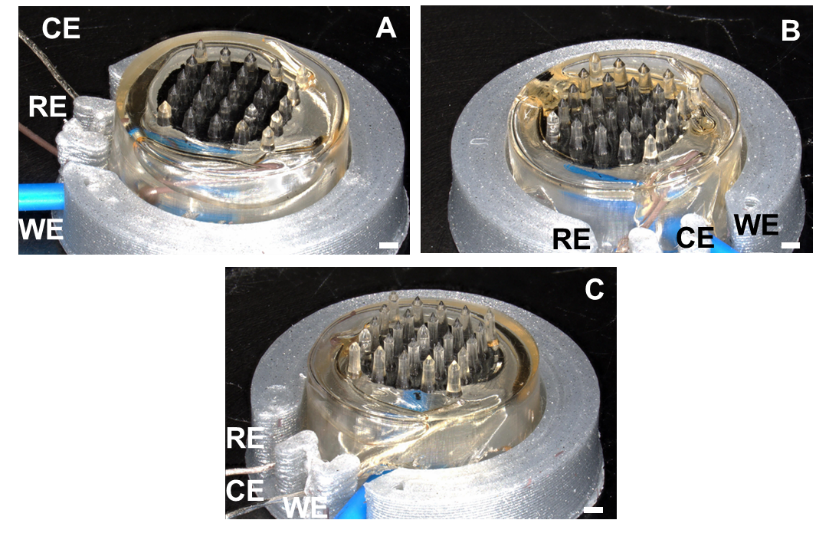


**Figure S22**. High resolution digital images of Dex-MA hydrogel MN arrays (DS = 62 %, 20 wt%) with integrated BP_PLQ_-GDH, Ag/AgCl wire, and Pt wire electrodes (MN-WE/RE/CE): (A) design A, (B) design B, and (C) design C. Scale bar = 500 µm. The 3D printed holder is shown in grey.

**Table S16**. Summary of the characteristic dimensions of Dex-MA hydrogel MN arrays (DS = 62 %, 20 wt%) with integrated BP_PLQ_-GDH, Ag/AgCl wire, and Pt wire electrodes (MN-WE/RE/CE), prepared with different MN designs. The data is presented as mean ± SD (*n =* 3).

| **Design** | **MN height**  **(µm)** | **MN base diameter**  **(µm)** | **MN top diameter (µm)** | **Inter-needle distance**  **(µm)** | **Base plate diameter**  **(µm)** |
| --- | --- | --- | --- | --- | --- |
| **A** | 779 ± 6 | 395 ± 2 | 324 ± 4 | 389 ± 3 | 6711 ± 22 |
| **B** | 966 ± 5 | 394 ± 3 | 323 ± 3 | 388 ± 3 | 6724 ± 31 |
| **C** | 1153 ± 8 | 394 ± 3 | 323 ± 3 | 386 ± 4 | 6717 ± 29 |

**Table S17.** Estimated electrochemical parameters and bioelectrocatalytic currents obtained from CVs recorded in 0.1 M PB for crosslinked Dex-MA MN-WE/RE/CE *vs.* MN-WE (DS = 62 %; 20 wt% polymer) prepared with different geometric areas. Steady-state bioelectrocatalytic currents obtained at 0.3 V *vs.* Ag/AgCl wire from CVs recorded at 1 mV s^-1^. The data is presented as mean ± SD (*n =* 3).

| Electrode | *E_1/2_* / V *vs.* Ag/AgCl wire | *ΔE_p_* / mV  (1 mV s^-1^) | *Γ* / mol cm^-2^  (20 mV s^-1^) | *Q* / *mF*  (20 mV s^-1^) | Current  density  (mA cm^-2^) |
| --- | --- | --- | --- | --- | --- |
| MN-WE  (DS = 62 %)  ø = 6 mm | -0.29 ± 0.02  -0.23 ± 0.03 | 42 ± 4  33 ± 4 | 1.54 ± 0.15 × 10^-7^ | 54.2 ± 3.6 | 0.26 ± 0.02 |
| MN-WE/RE/CE  (DS = 62 %)  ø = 4 mm | -0.30 ± 0.02  -0.22 ± 0.01 | 39 ± 3  34 ± 2 | 1.49 ± 0.15 × 10^-7^ | 20.9 ± 1.3 | 0.29 ± 0.03 |

**Table S18.** Estimated electrochemical parameters and bioelectrocatalytic current obtained from CVs recorded in artificial ISF-containing artificial soft skin for crosslinked Dex-MA MN-WE/RE/CE (DS = 62 %; 20 wt% polymer). Steady-state bioelectrocatalytic currents obtained at 0.3 V *vs.* Ag/AgCl wire from CVs recorded at 1 mV s^-1^. The data is presented as mean ± SD (*n =* 3).

| Electrode | *E_1/2_* / V *vs.* Ag/AgCl wire (V) | *ΔE_p_* / mV  (1 mV s^-1^) | *Γ* / mol cm^-2^  (20 mV s^-1^) | *Q* / *mF*  (20 mV s^-1^) | Current  density  (mA cm^-2^) |
| --- | --- | --- | --- | --- | --- |
| MN-WE/RE/CE  (DS = 62 %) | -0.18 ± 0.01  -0.07 ± 0.01 | ≤ 36 ± 4  31 ± 2 | 1.61 ± 0.13 × 10^-7^ | 21.9 ± 1.4 | 0.24 ± 0.03 |


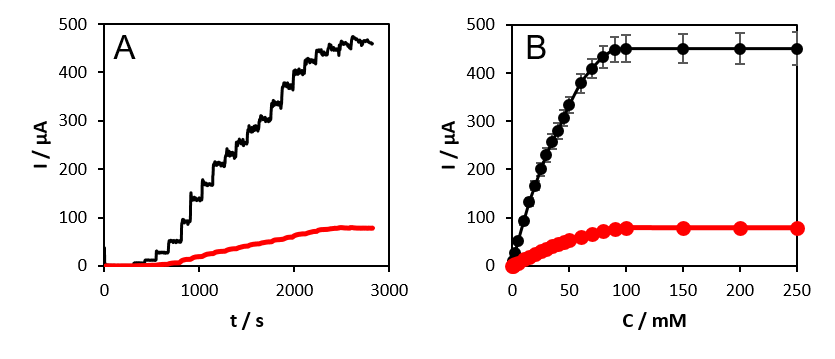


**Figure S23**. Fixed potential chronoamperograms recorded at (black) WE (BP_PLQ_-GDH, ø = 4 mm) and (red) MN-WE/RE/CE (ø = 4 mm) at increasing glucose concentrations at an applied potential of -0.1 V *vs.* Ag/AgCl at 500 rpm. Bioelectrocatalytic oxidation current *vs.* concentration plotted from the corresponding fixed potential CA measurements. The data obtained at BP_PLQ_-GDH is presented as mean ± SD (*n =* 3).

**Table S19**. Characteristic analytical biosensor parameters for WE (BP_PLQ_-GDH) and MN-WE/RE/RE (DS = 62%) extracted from fixed potential CA measurements recorded at an applied potential of -0.1 V *vs.* Ag/AgCl at 500 rpm. The data is presented as mean ± SD (*n =* 3).

| **Electrode** | **Linear range**  **(mM) ; r^2^** | **Sensitivity**  **(µA mM^-1^ cm^-2^)** | **Limit of detection**  **(mM)** | **Stabilisation time**  **(s)** |  |
| --- | --- | --- | --- | --- | --- |
| WE  ø = 4 mm | 0.05 – 25 ; 0.995 | 66.7 ± 4.0 | 0.1 | 26.9 ± 4.2 |  |
| MN-WE/RE/CE  (DS = 62 %)  ø = 4 mm | 0.05 – 50 ; 0.995 | 10.1 ± 0.9 | 0.1 | 100.3 ± 7.8 | |

**Table S20**. Characteristic biosensor calibration parameters for the MN-WE/RE/RE (DS = 62%) in artificial soft skin. The values were estimated at different glucose concentrations using calibration methods based on (i) the raw current output or (ii) the decay slope. The values were obtained from fixed potential CA measurements recorded at an applied potential of -0.1 V *vs.* Ag/AgCl. The data is presented as mean ± SD (*n =* 3).

|  | **1 mmol L^-1^** | **5 mmol L^-1^** | **10 mmol L^-1^** | **25 mmol L^-1^** |
| --- | --- | --- | --- | --- |
| **I max (µA)** | 0.21 ± 0.03 | 1.12 ± 0.10 | 2.11 ± 0.19 | 5.33 ± 0.21 |
| **Slope (µA jour^-1^) ; R^2^** | - 0.028 ± 0.007 ; 0.972 | - 0.134 ± 0.016 ; 0,986 | - 0.314 ± 0.022 ; 0.982 | - 0.810 ± 0.062 ; 0.985 |


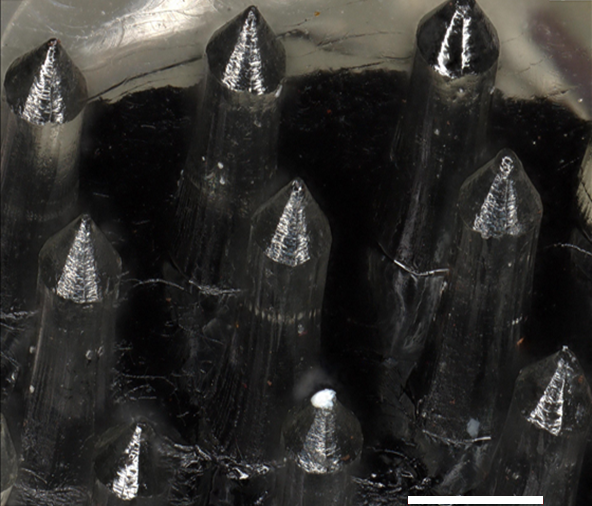


**Figure S24.** High resolution digital images of Dex-MA hydrogel MN arrays (DS = 62 %, 20 wt%) after 10 days of CGM in the hard skin model.

**References**

[1] P. Makvandi, M. Kirkby, A. R. J. Hutton, M. Shabani, C. K. Y. Yiu, Z. Baghbantaraghdari, R. Jamaledin, M. Carlotti, B. Mazzolai, V. Mattoli, R. F. Donnelly, *Nanomicro Lett.* **2021**, *13*, 93.

[2] H. Chang, M. Zheng, X. Yu, A. Than, R. Z. Seeni, R. Kang, J. Tian, D. P. Khanh, L. Liu, P. Chen, C. Xu, *Adv. Mater.,* **2017**, 29, 37, 1702243

[3] A. Sadeqi, G. Kiaee, W. Zeng, H. Rezaei Nejad, S. Sonkusale, *Sci. Rep.* **2022**, *12*, 1853.

[4] P. P. Samant, M. M. Niedzwiecki, N. Raviele, V. Tran, J. Mena-Lapaix, D. I. Walker, E. I. Felner, D. P. Jones, G. W. Miller, M. R. Prausnitz, *Sci. Transl. Med.* **2020**, *12*, eaaw0285.

[5] P. P. Samant, M. R. Prausnitz, *Proc. Natl. Acad. Sci. U.S.A.* **2018**, *115*, 4583.

[6] A. I. Chen, M. L. Balter, M. I. Chen, D. Gross, S. K. Alam, T. J. Maguire, M. L. Yarmush, *Med. Phys.* **2016**, *43*, 3117.

[7] H. R. Luthfianti, W. X. Waresindo, D. Edikresnha, A. Chahyadi, T. Suciati, F. A. Noor, K. Khairurrijal, *ACS Omega* **2023**, *8*, 2915.

[8] W. R. Kennedy, G. Wendelschafer-Crabb, *J. Neurol. Sci.* **1993**, *115*, 184.

[9] M. Geerligs, L. van Breemen, G. Peters, P. Ackermans, F. Baaijens, C. Oomens, *J. Biomech.* **2011**, *44*, 1176.

[10] C. Lorenz, W. Sandoval, M. Mortellaro, *Diabetes Technol. Ther.* **2018**, *20*, 344.

[11] W.-Y. Liao, T.-C. Chou, *Anal. Chem.* **2006**, *78*, 4219.

[12] N. Fogh-Andersen, B. M. Altura, B. T. Altura, O. Siggaard-Andersen, *Clin. Chem.* **1995**, *41*, 1522.

[13] M. I. Mackness, B. Mackness, S. Arrol, G. Wood, D. Bhatnagar, P. N. Durrington, *FEBS Lett.* **1997**, *416*, 377.

[14] A. L. Krogstad, P. A. Jansson, P. Gisslén, P. Lönnroth, *Br. J. Dermatol.* **1996**, *134*, 1005.

[15] L. Strindberg, P. Lönnroth, *Scand. J. Clin. Lab. Invest.* **2000**, *60*, 205.

[16] A. Heller, B. Feldman, *Chem. Rev.* **2008**, *108*, 2482.
